# Intravenous magnesium sulfate for the management of severe hand, foot, and mouth disease with autonomic nervous system dysregulation in Vietnamese children: study protocol for a randomized controlled trial

**DOI:** 10.1186/s13063-016-1215-6

**Published:** 2016-02-19

**Authors:** Phan Tu Qui, Truong Huu Khanh, Huynh Trung Trieu, Phạm Thanh Giang, Nguyen Ngọc Bich, Le Phan Kim Thoa, Le Nguyen Thanh Nhan, Saraswathy Sabanathan, Rogier Van Doorn, Nguyen Duc Toan, Laura Merson, Nguyen Thi Phuong Dung, Lam Phung Khanh, Marcel Wolbers, Nguyen Thanh Hung, Nguyen Van Vinh Chau, Bridget Wills

**Affiliations:** Hospital for Tropical Diseases, 764 Vo Van Kiet, District 5, Ho Chi Minh City, Vietnam; Oxford University Clinical Research Unit, Hospital for Tropical Diseases, 764 Vo Van Kiet, Quan 5, Ho Chi Minh City, Vietnam; Children’s Hospital Number 1, 341 Sư Vạn Hạnh, District 10, Ho Chi Minh City, Vietnam; Centre for Tropical Medicine and Global Health, Nuffield Department of Clinical Medicine, Oxford University, Oxford, OX1 2JD UK

**Keywords:** Hand, foot, and mouth disease, brainstem encephalitis, magnesium sulfate, randomized controlled trial

## Abstract

**Background:**

Over the last 15 years, hand, foot, and mouth disease (HFMD) has emerged as a major public health burden across the Asia-Pacific region. A small proportion of HFMD patients, typically those infected with enterovirus 71 (EV71), develop brainstem encephalitis with autonomic nervous system (ANS) dysregulation and may progress rapidly to cardiopulmonary failure and death. Although milrinone has been reported to control hypertension and support myocardial function in two small studies, in practice, a number of children still deteriorate despite this treatment. Magnesium sulfate (MgSO_4_) is a cheap, safe, and readily available medication that is effective in managing tetanus-associated ANS dysregulation and has shown promise when used empirically in EV71-confirmed severe HFMD cases.

**Methods/Design:**

We describe the protocol for a randomized, placebo-controlled, double-blind trial of intravenous MgSO_4_ in Vietnamese children diagnosed clinically with HFMD plus ANS dysregulation with systemic hypertension. A loading dose of MgSO_4_ or identical placebo is given over 20 min followed by a maintenance infusion for 72 h according to response, aiming for Mg levels two to three times the normal level in the treatment arm. The primary endpoint is a composite of disease progression within 72 h defined as follows: development of pre-specified blood pressure criteria necessitating the addition of milrinone, the need for ventilation, shock, or death. Secondary endpoints comprise these parameters singly, plus other clinical endpoints including the following: requirement for other inotropic agents; duration of hospitalization; presence of neurological sequelae at discharge in survivors; and neurodevelopmental status assessed 6 months after discharge. The number and severity of adverse events observed in the two treatment arms will also be compared. Based on preliminary data from a case series, and allowing for some losses, 190 patients (95 in each arm) will allow detection of a 50 % reduction in disease progression with 90 % power at a two-sided 5 % significance level.

**Discussion:**

Given the large numbers of HFMD cases currently being seen in hospitals in Asia, if MgSO_4_ is shown to be effective in controlling ANS dysregulation and preventing severe HFMD complications, this finding would be important to pediatric care throughout the region.

**Trial registration:**

ClinicalTrials.gov Identifier: NCT01940250 (Registered 22 August 2013).

**Electronic supplementary material:**

The online version of this article (doi:10.1186/s13063-016-1215-6) contains supplementary material, which is available to authorized users.

## Background

Hand, foot, and mouth disease (HFMD) is a common infectious disease, primarily affecting young children, caused by a number of enteroviruses belonging to the species *Human enterovirus A* and including coxsackievirus a16 (CA16) and enterovirus 71 (EV71). Infection with EV71 is of particular concern because it can cause severe HFMD, sometimes resulting in death. Over the past 15 years, EV71-associated HFMD has emerged across Asia, with outbreaks thought to involve millions of people occurring in the region [1]. EV71-related HFMD has also increased markedly in Vietnam during this period, and in 2011, 170 deaths occurred among the 113,000 Vietnamese children clinically diagnosed with the disease [2].

### Autonomic nervous system (ANS) dysregulation and HFMD

During outbreaks, thousands of children can develop HFMD, and although most will have self-limited illness with fever and rash only, a small proportion will develop neurological and systemic complications that can be rapidly fatal. Neurological manifestations of EV71 infection include aseptic meningitis and acute flaccid paralysis, but the issue of most concern is brainstem encephalitis because autonomic nervous system (ANS) dysregulation may occur, potentially with rapid progression to cardiopulmonary failure [1]. Clinical features indicating ANS dysregulation include high persistent fever, profuse sweating, mottled skin, tachycardia, tachypnoea, hypertension, and hyperglycemia [3]. The Vietnamese Ministry of Health (MoH) has developed guidelines for clinical staging and suggested management of HFMD, according to the severity grade (Additional file 1: Appendix 1). Briefly, Grade 1 represents classic HFMD without complications. Patients with Grade 2 disease show some evidence of central nervous system involvement, usually manifesting as myoclonus. In Grade 3 disease, evidence of ANS dysfunction is present, whereas patients with Grade 4 disease have cardio-pulmonary compromise.

Although the mechanisms underlying the ANS dysregulation have not been clearly defined, evidence exists that inflammation occurs in the medulla oblongata and cervical spinal cord, causing increased sympathetic activity and resulting in severe systemic and pulmonary hypertension and eventually pulmonary edema [1]. The frequency of ANS dysregulation is also unclear because many of the signs are rather subjective and also can be present among children with high fever; however, the development of systemic hypertension in children with HFMD is considered to be unequivocal evidence of ANS dysregulation. The frequency with which this occurs is not known, however, because only severe cases are usually reported. In one study of 36 severe EV71 infection cases from Taiwan, more than 36 % developed systemic hypertension, whereas in 22 severe HFMD cases reported from China, 17 cases had hypertension [4].

Research has also revealed left ventricular dysfunction in patients with EV71-associated brainstem encephalitis/hypertension who later develop pulmonary edema. Although no histological or virological evidence of viral myocarditis was seen, catecholamine-associated cardiotoxic effects were found on histological examination of cardiac ventricular biopsies, together with high concentrations of norepinephrine and epinephrine in plasma from these patients [5]. Thus, high plasma catecholamine concentrations secondary to brainstem encephalitis are purported to have a direct effect on cardiac function, as well as to cause pulmonary edema by raising pulmonary pressures [1]. Alternatively, or in addition, the impact of altered cytokine profiles on the cardiopulmonary system may influence the severity of HFMD [6]. In small studies, the severity of EV71 infection has been associated with altered concentrations of a number of different cytokines, including TNF∝, INFγ, IL6, and IL13, in blood and cerebrospinal fluid (CSF), with evidence that cytokine levels correlate with the degree of injury to the brainstem and spinal ganglia [7]. Moreover, in a number of animal studies of neurogenic hypertension, associations have been demonstrated between inflammatory cytokine levels and enhanced vasomotor and cardiac sympathetic drive [8].

### Management of ANS dysregulation

Management of ANS dysregulation presents particular challenges. In one report from Taiwan, use of the phosphodiesterase-3 inhibitor, milrinone was said to control hypertension and support myocardial function in a group of 24 children with severe HFMD compared to historical controls [9]. In addition, milrinone was shown to decrease mortality in HFMD patients with pulmonary edema in a small, open-label, randomized clinical trial in Vietnam [10]. On the basis of these small studies, milrinone has now become the recommended therapy for HFMD with ANS dysregulation in Vietnam, with MoH guidelines setting down indications for when the drug should be commenced in suspected HFMD cases. Currently, the MoH guidelines define the intervention level for use of milrinone to be when the systolic blood pressure (SBP) exceeds the 99th percentile for age plus 5 mmHg, which approximates to the internationally accepted definition of Stage 2 hypertension in children (Additional file 1: Appendix 1) [11]. However, clinical failures still occur despite high dose intravenous milrinone, and a number of children go on to require hemofiltration and ventilatory support, the next steps recommended in the Vietnamese MoH guidelines (Additional file 1: Appendix 2). Secondly, there are few clinical or safety data available with respect to milrinone use in children, apart from a few small studies following cardiac surgery [12] [13]. Recent reports suggest that milrinone use is an independent risk factor for clinically significant tachyarrhythmia after congenital heart surgery, and may be associated with development of acute renal failure [13].

### Magnesium sulfate and ANS dysregulation

Autonomic disinhibition is also postulated to occur in severe tetanus. Elevated concentrations of circulating catecholamines have been observed, and urinary epinephrine and norepinephrine excretion are increased proportional to disease severity [14, 15]. In a randomized controlled trial comparing magnesium sulfate (MgSO_4_) with placebo in patients with severe tetanus, the use of magnesium was associated with significantly reduced requirements for drugs to control muscle spasms and cardiovascular instability [16]. In patients undergoing tracheal intubation or surgery for pheochromocytoma, the use of magnesium was associated with a reduction in SBP and in plasma catecholamine concentrations [17]. Isolated reports exist of rapid and effective control of life-threatening autonomic hyperreflexia in patients with spinal cord lesions [18–22]. In in vitro studies, magnesium has been shown to reduce catecholamine secretion from the peripheral nerve endings and the adrenal medulla [23]. Intrapartum use of MgSO_4_ in women with preeclampsia was associated with reduced cytokine levels in the women and their babies [24]. Similarly, the use of magnesium in a small number of patients with aneurysmal subarachnoid hemorrhage was associated with reduced serum levels of certain inflammatory cytokines [25]. Formal safety data relating to the use of MgSO_4_ in pediatric care are limited, but from its use in children with severe asthma [18] and in a small number of neonates with uncontrolled pulmonary hypertension [26], adverse effects appear to be infrequent.

In summary, although HFMD has become one of the major contributors to childhood morbidity and mortality in Vietnam, current management strategies rely on guidelines that are based on expert opinion and only two small clinical studies. Vietnamese MoH guidelines currently indicate that milrinone should be used when Stage 2 hypertension develops, but evidence for the efficacy of this intervention is limited, and a number of children (approximately 20 %, personal communication Dr. Phan Tu Qui) treated in this way require additional interventions. In addition, milrinone is an expensive drug with a known toxicity profile and requires highly experienced staff to administer it. We hypothesized that early intervention with MgSO_4_ in Grade 3 HFMD, when Stage 1 hypertension and ANS dysregulation first become apparent, might control cardiovascular instability more effectively and prevent progression to more severe disease. MgSO_4_ is cheap, readily available, and generally considered to be safe. Given the very large numbers of HFMD cases currently being seen in hospitals in Asia, if MgSO_4_ is shown to be effective in controlling ANS dysregulation and preventing severe HFMD complications, this finding would be important to pediatric care throughout the Asian region.

#### Primary objective of the trial

The primary objective of the trial is to evaluate the effects of MgSO_4_ for the control of hypertension and progression to severe disease (shock, respiratory compromise, or death) in children with severe HFMD and ANS dysregulation.

#### Secondary objectives of the trial

The secondary objectives of the trial are as follows:

1. To describe the clinical signs of autonomic dysfunction observed in children with severe HFMD; measure levels of biomarkers of sympathetic activity, including plasma and urine catecholamine levels and levels of inflammatory cytokines; and to assess the impact of MgSO_4_ on these parameters.
2. To examine the relations between measures of cardiac output (CO) and systemic vascular resistance (SVR) with clinical signs of autonomic dysfunction and to assess the impact of MgSO4 on these parameters.
3. To evaluate the effects of MgSO_4_ on long-term outcome from severe HFMD in survivors, by assessing neurodevelopmental status at 6 months.

## Methods/Design

### Study Design

This is a randomized, placebo-controlled, double-blind trial to assess the efficacy and side effect profile of intravenous MgSO_4_ when used in Vietnamese children with clinically diagnosed HFMD and signs of ANS dysregulation with systemic hypertension.

### Study sites

The Hospital for Tropical Diseases (HTD) and Children’s Hospital Number One (CH1) are two major hospitals in Ho Chi Minh City, Vietnam, with 1600 in-patient beds in total. Each hospital functions as a first-level facility for the immediate local population and as a tertiary referral center responsible for the diagnosis and management of serious infectious diseases for a population of over 15 million children across southern Vietnam. The Oxford University Clinical Research Unit (OUCRU) is located on the HTD site, and together, the three institutions have established a number of fruitful research collaborations over more than 20 years.

### Inclusion and exclusion criteria (see Additional file 1: Appendices 1-4 for definitions/additional details)

Vietnamese MoH guidelines indicate that all suspected HFMD cases with Grade 2 or more severe disease should be admitted to a Pediatric Intensive Care Unit (PICU) or High Dependency Unit (HDU) facility for close observation. The guidelines also indicate that a peripheral arterial line should be inserted for invasive BP monitoring if there are any signs of ANS dysfunction. For patients with Grade 3 disease (that is, the group eligible for inclusion in this study), the pulse, BP, respiratory rate and pattern, oxygen saturation, and temperature must be monitored very closely, and the child should receive intravenous sedation with phenobarbitone, as well as a dose of 1 g/kg intravenous immunoglobulin (IVIG) as soon as possible, with a second dose of IVIG 24 h later.

All patients aged 6 months to 15 years admitted to the PICU at HTD or the HDU on the Infectious Diseases Ward at CH1 in Ho Chi Minh City with clinically suspected HFMD (Additional file 1: Appendix 1) are considered for eligibility for this trial. Patients are eligible for enrollment if a) the arterial blood pressure (BP), measured via an indwelling catheter, exceeds the internationally recognized definition for Stage 1 hypertension in children (Additional file 1: Appendix 3) [27]; b) they exhibit at least one other criterion for ANS dysregulation (Additional file 1: Appendix 4), such as tachypnea for age, irregular or labored breathing but with oxygen saturation above 92 % in air and a normal arterial blood gas, resting heart rate sustained above 150 beats/minute, mottled skin, profuse sweating, refractory fever, or hyperglycemia; and c) no contraindications to study inclusion are present, including a past history of hypertension, chronic renal, cardiac or pulmonary disease, or any neurological disorder; features indicating a current hypertensive emergency (see below); treatment with milrinone or any other inotropic agent has already commenced; respiratory distress is present with oxygen saturation below 92 % in air or an arterial pCO_2_ over 45 mmHg; atrioventricular block or any arrhythmia (other than sinus tachycardia) is present on an ECG rhythm strip, or the QT interval is prolonged; or reduced urine output or increased creatinine levels indicate renal compromise. If the patient fulfils these criteria and a parent or guardian gives written informed consent (see “Ethical Considerations” section), then the child is enrolled in the study.

The current Vietnamese MoH guidelines specify that milrinone should be given to children with HFMD and ANS dysregulation when the SBP is sustained at a level exceeding a value approximating the 99th percentile for age plus 5 mmHg (that is, Stage 2 hypertension) with the intention that treatment should commence within 1 to 2 h [28]. Enrollment to this study is designed to be early, when hypertension is first identified (at Stage 1), so that in the event of treatment failure, the MoH treatment guidelines can be applied. A small number of patients who present with Stage 2 hypertension may also be enrolled; in such cases, very stringent BP criteria will ensure that, if the BP does not improve within 30 min of commencing the study drug, milrinone will be added. That is, all Stage 2 patients will be on milrinone within 1 h of presentation unless the BP falls to Stage 1 levels.

### Intervention / study medication

Detailed information on the use of MgSO_4_, the pharmacokinetics, the side effects profile, etc. is provided in Additional file 1: Appendix 5. Patients are assigned to one of two treatment arms and follow the same dosing schedule in both arms. After written informed consent has been obtained, staff administer a loading dose of 50 mg/kg of either MgSO_4_ or a visually matched placebo (sterile water) by continuous infusion into a peripheral intravenous line over 20 min, followed by a maintenance infusion in the treatment arm of 30 to 50 mg/kg/h for 72 h according to response, aiming for plasma Mg levels two to three times the normal level. All staff members involved in the clinical care remain blind to the treatment allocation, and Mg levels are monitored and adjusted by independent doctors from another clinical facility as detailed in the section on “dose adjustment” below.

### Study endpoints

*The primary endpoint* is a composite endpoint indicating disease progression within 72 h. It is defined as the occurrence of any of the following within 72 h of commencing the study drug infusion:

1. Specific BP criteria necessitating the addition of milrinone as detailed in the section “Criteria for the addition of milrinone” below.
2. Need for mechanical ventilation.
3. Development of shock.
4. Death.

*Secondary endpoints* comprise these parameters singly, plus a number of other clinical endpoints including the following: a requirement for other inotropic agents (for example, dobutamine), duration of hospitalization, presence of neurological sequelae at discharge in survivors, and neurodevelopmental status assessed 6 months after discharge.

*Safety endpoints* include the number of adverse events (AEs) and severe adverse events (SAEs) that occur in the two treatment arms.

*Exploratory endpoints* will be assessed, including serial measurements of cardiac output (CO) and systemic vascular resistance (SVR), catecholamine levels, and cytokine levels. We will assess the impact of MgSO_4_ on these parameters, provided the data collected appear reliable and consistent within individual participants.

### Criteria for the addition of milrinone

Milrinone will be commenced in any of the following circumstances to ensure compliance with the Vietnamese MoH guidelines for management of HFMD:

1. Hypertensive emergency, which includes a severe symptomatic elevation in BP (>30 % compared to baseline BP) WITH evidence of acute target organ damage, for example, brain (seizures or increased intracranial pressure), kidneys (renal insufficiency), eyes (papilledema, retinal hemorrhages, or exudates), or heart (heart failure).
2. For children aged 1 year and older:
  - SBP increases to ≥ 99th percentile plus between 5 and 15 mmHg consistently for 30 min.
  - SBP increases to ≥ 99th percentile plus 15 mmHg consistently for 15 min
  - SBP increases to ≥ 40 mmHg over baseline for 15 min and this value is lower than either of the first two cut-offs. The baseline SBP is defined as the lowest value measured at any time after admission to hospital before enrollment in the study.
3. For children aged 6 months to 1 year:
  - SBP increases to ≥ 110 mmHg up to 120 mmHg consistently for 30 min.
  - SBP increases to ≥ 120 mmHg consistently for 15 min.

Occasionally, the clinical status of a patient may indicate that milrinone is not the best inotrope to use. In these cases, the treating physician will make management decisions appropriate to the situation. Similarly, if the patient’s clinical status remains unstable after starting milrinone, additional measures, including ventilation and/or hemofiltration, will be considered in accordance with Vietnamese MoH guidelines for management of HFMD (Additional file 1: Appendix 2). If the BP remains high (SBP > 99th percentile plus > 15 mmHg) despite maximal doses of milrinone and study drug infusion, then additional antihypertensive agents may be added, such as nicardipine, captopril, etc. depending on the clinical scenario.

### Study procedures

#### Screening and recruitment of participants (Fig. 1)

**Fig. 1:**
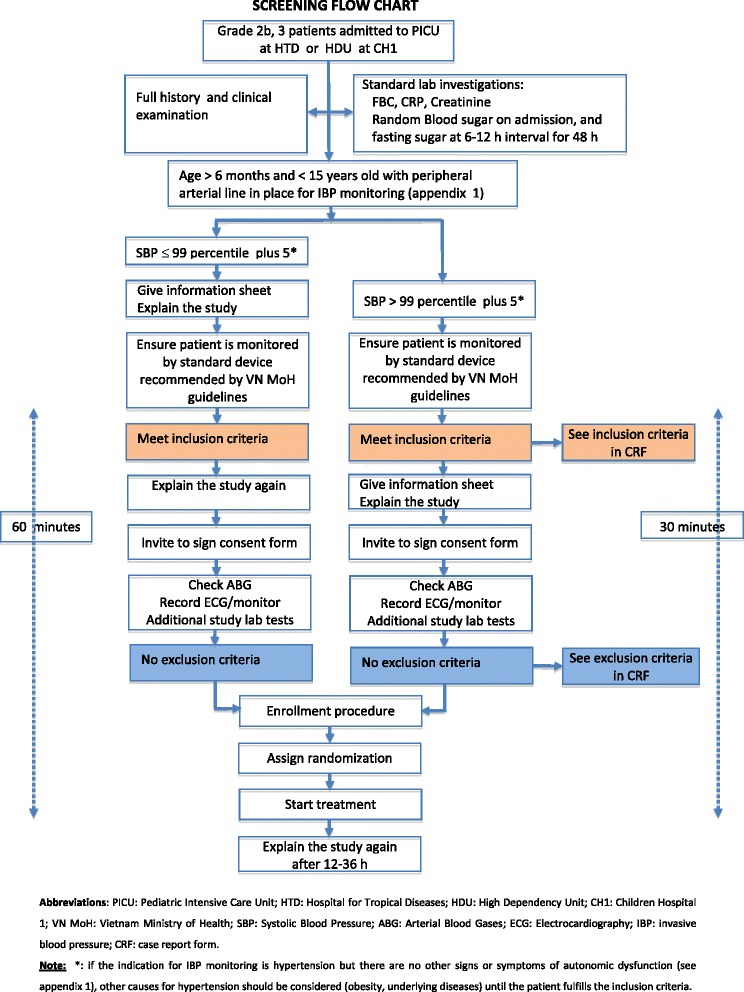
Screening flow chart

##### Phase 1 (HTD only)

Initially, recruitment is scheduled to take place at one site (HTD) only to ensure that all study procedures run smoothly during the first few months. Study staff working on PICU at HTD will identify parents or guardians of potentially eligible patients as soon as possible after admission. They consult the hospital chart and the parents/guardians to verify if the initial screening criteria are met, then provide the patient information sheet (PIS, see Additional file 1: Appendix 7) and discuss the study with relevant families. This allows time for the family members to consider the study without pressure to decide immediately. A reference card is completed at this time, defining the specific intra-arterial BP thresholds needed for study enrollment, the addition of milrinone, treatment failure, etc. relevant to that particular individual (by age, sex and length), and this card is attached to the child’s observation chart at the bedside. If the child subsequently develops autonomic disturbance with hypertension exceeding the defined threshold, the study staff go through the PIS a second time with the family and answer any questions before asking for consent, then follow the full pathway for inclusion/exclusion criteria, and if appropriate, proceed to enrollment and randomization (Fig. 1). A case report form (CRF) detailing the history and examination findings at enrollment is completed as soon as the study drug infusion has been started. Children whose parents/guardians do not consent to the study continue with standard care.

Occasionally, patients may present with established Stage 2 hypertension; these individuals have arterial access established immediately, and the process of explaining the study, requesting consent, checking inclusion/exclusion criteria and proceeding to enrolment and randomization, if appropriate, is carried out as quickly as possible, with the aim of commencing the study drug within 30 min. If the BP does not improve within 30 min of commencing the study drug, the milrinone is added. That is, all Stage 2 patients will be on milrinone within 1 h of presentation unless the BP has settled to Stage 1 levels with the study drug.

In case worry over the child’s illness could affect the parents/guardians ability to make an informed decision on study participation, a study doctor goes though the PIS with the family of all participants at least once more in the 12 to 36 h after enrollment or at any time they ask. This review of the PIS is recorded in the CRF.

##### Phase 2 (HTD and CH1)

The study will be established and executed in a similar manner on the HDU at CH1 in due course, with both sites aiming to complete the recruitment over 2 to 3 years.

#### Randomization and blinding

Randomization follows a 1:1 ratio, stratified according to the hospital where recruitment takes place. A randomization list using block randomization with blocks of variable size has been prepared by the Clinical Trial Pharmacist using a computer program and is maintained confidentially from all study staff and treating doctors, including those assessing all trial outcomes. A chronological log of all enrolled patients is maintained by the Pharmacist and the next available sequential study code is assigned to each patient as they enroll. The assigned number corresponds to a coded, sealed, package containing 50 ampoules of 15 % MgSO_4_ or visually matched placebo.

#### Dispensing, storage and accountability

MgSO_4_ 15 % solution and placebo (sterile water) are available in 10 ml visually matched ampoules supplied by Fresenius Kabi. Study drug packages are prepared centrally by the un-blinded Clinical Trials Pharmacist and distributed to the sites as required. Drugs are stored in accordance with the manufacturers’ recommendations in a secure area. All movements of study medication are recorded. Both individual subject and overall drug accountability records are kept up to date. Prior to administration each vial is diluted with 5 ml 0.9 % saline solution by trained study nurses, to give a volume of 15 ml and provide a 10 % MgSO_4_ solution for the magnesium intervention arm.

#### Study drug dose adjustment (Fig. 2)

**Fig. 2:**
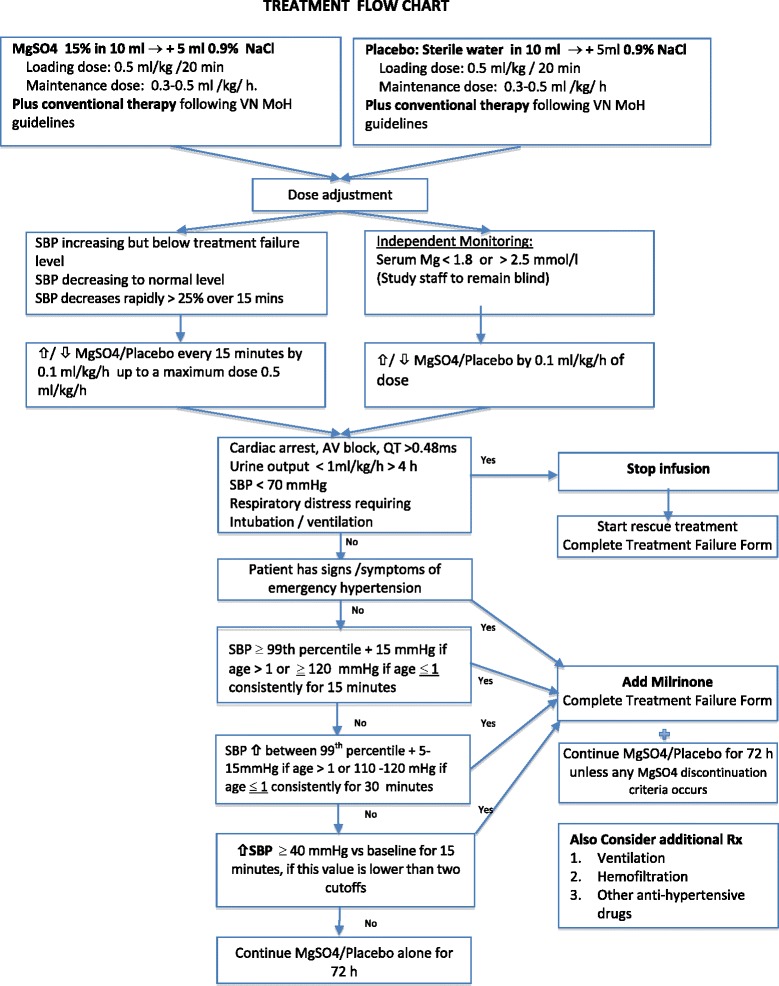
Treatment flow chart

After the initial loading dose, the study infusion dose is increased in 0.1 ml/kg/h stages (10 mg/kg/h) every 15 min to a maximum dose of 0.5 ml/kg/h (50 mg/kg/h), with the following caveats:

- If the SBP decreases to < 90th percentile for age, sex, and length, the study drug infusion is reduced by one stage every 15 min.
- If the SBP increases to the levels described below for treatment failure, action is taken as indicated.
- Plasma Mg levels are measured at least once daily. If the plasma Mg level is > 2.5 mmol/l or < 1.8 mmol/l, an increase or decrease in the infusion rate by 0.1 ml/kg/h is implemented as appropriate. To maintain blinding, plasma Mg values are communicated by the laboratory staff to an independent doctor not involved in the clinical care of the patient, who informs the clinical staff of any dose adjustments to be made to the study drug infusion but does not report the actual laboratory values. Similar (sham) dose adjustments are made for the placebo infusions according to a randomized list available only to the independent doctor. However, the clinicians managing the patient may request unblinding at any time.

#### Discontinuation of the study treatment

If any of the following occur, the study treatment is stopped immediately, and rescue treatment is given as appropriate (Additional file 1: Appendix 5, 6):

1. Serious cardiac arrhythmia (for example, atrio-ventricular block, prolonged QT interval)
2. Hypotension: SBP < 70 + (2 × age) mmHg for 15 min or more
3. Urine output < 1 ml/kg/h for 4 h or more
4. Cardiac arrest or any other emergency situation where the treating physician feels there is a contra-indication to the study drug.

In addition, if a patient develops respiratory distress (as defined in Additional file 1: Appendix 4), an urgent plasma Mg level will be performed. If intubation and ventilation is needed or if the Mg level is > 3 mmol/l, the study treatment will be stopped, and rescue treatment will be given as described in Additional file 1: Appendix 6.

#### Unblinding

The Clinical Trials Pharmacist holds the unblinded randomization list with details of the contents of each individual treatment package. This list will be accessed only in the case of emergency unblinding, which will be authorized by an investigator following standard operating procedures. Emergency unblinding will be performed in the case of an adverse event when knowledge of the identity of the study treatment may contribute to the treating physician’s ability to care for the patient. In this case, the treating doctor will confirm with the Principal Investigator that unblinding may help inform the care of the patient. If agreed on by the Principal Investigator, the treating doctor may request to know the Mg/Ca lab results or may open the sealed emergency envelopes available on the ward that contain individual patient treatment allocations. In general, in these cases, the study treatment would already have been discontinued as per the “*Discontinuation of study treatment*” paragraph.

#### Clinical assessments and follow-up (Additional file 1: Appendix 8)

All participants remain in the study for the duration of follow-up to 6 months unless the parent/guardian wishes to withdraw, which they may do at any time (see withdrawal section, page 28). In addition to the study procedures outlined below, all patients will receive standard care according to the national treatment guidelines. During the acute phase, patients are examined daily by trained HDU/PICU study doctors. A record of all significant events in the previous 24 h plus detailed physical examination findings (in particular focusing on the nervous system, respiratory, and cardiovascular systems) is recorded each morning in the specially designed CRF. Vital signs are monitored continuously using devices with the facility for download and recording of data into a readable database format using compatible software and/or accessory modules. In a subset of patients, advanced hemodynamic parameters such as CO and SVR are recorded using LiDCO monitoring systems, the subset being determined by the availability of the LiDCO technology at the time when an individual is enrolled and randomized.

A standard ECG recording is performed at the bedside by a trained nurse once daily and also if any abnormality is noted on the monitor by study staff (for example, premature beats, or atrial or ventricular arrhythmias). Urine output is monitored using specific collecting bags and calculated every 4 h. If the urine output is less than 1 ml/kg/h over a 4-h period, a bedside ultrasound scan is done, and if necessary, urethral catheterization is established to monitor the urine output more closely.

Magnesium is an important cofactor in many enzymatic reactions in the body, but the side effect profile is well known, and in the dose range used here, it is generally considered safe (Additional file 1: Appendix 5). However, differentiation between potential magnesium toxicity and progression of HFMD can be difficult. All staff involved in the study have been trained to recognize potential side effects, and a series of standard operating procedures have been put in place indicating when to stop study treatment and/or initiate rescue therapy if any complications occur that might be related to the treatment (Additional file 1: Appendix 6). Similarly, all staff members are aware that they may request an urgent Mg/Ca level at any time or request emergency unblinding in the case of any adverse event when knowledge of the identity of the study treatment might contribute to the treating physician’s ability to care for the patient.

Patients are assessed daily for the duration of the hospital stay. At discharge, full neurological and neurodevelopmental assessments are performed. Patients who have not recovered fully from the effects of sedation with phenobarbitone by the time of discharge, but are otherwise considered fit to go home, are asked to attend 1 week later for formal review and neurological assessment. All patients are also asked to return at 6 months post-enrollment for a clinical and neurodevelopmental assessment. After this, any further follow-up will be according to clinical need, and participants will be referred back to the standard hospital outpatient clinic system.

Neurodevelopmental assessments done at discharge (or 1 week later) and 6 months use the Bayley Scales of Infant Development, Third Edition (Bayley-III) and Movement ABC-2 tools. Children 36 months and under at enrollment will use the Bayley-III. Children 48 months and above at enrollment will use the Movement ABC-2 tool for their assessments.

#### Laboratory assessments and diagnostic testing

The following laboratory tests (see Additional file 1: Appendix 9) are performed on site in the standard hospital laboratories or at the research laboratory of OUCRU in Ho Chi Minh City, except where specifically indicated below:

1. Blood glucose and arterial blood gases are measured at least once daily or more frequently according to the clinical situation.
2. Plasma magnesium and calcium concentrations are measured at baseline and 12 h after the start of the study infusion, then once daily for the remaining 72 h.
3. Plasma electrolytes, creatinine, CKMB and Troponin I are measured at baseline and once daily thereafter.
4. Samples for plasma catecholamine (epinephrine and norepinephrine) concentrations are collected at baseline and then once daily for 72 h, and stored frozen for subsequent analysis in batches. Similarly, a 5-ml aliquot of urine is collected each morning for measurement of urine catecholamines. All urine is collected in appropriate collecting bottles (containing 10 to 15 ml 6 M HCl as preservative) by the bedside and the total volume is recorded at the end of the 24-h period, before the 5-ml aliquot is taken for storage at −20 °C.
5. Samples for plasma cytokine measurements (including IL1, IL6, IL13, and TNF alpha) are collected and stored at −20 °C at baseline and at 12 h and 24 h after starting the MgSO_4_/placebo, with a final sample obtained on discharge from hospital.
6. A nasal/throat swab and a rectal swab are obtained at enrollment, for enterovirus PCR.
7. Serological testing for EV71 and related serotypes within the *Human enterovirus A* species will be done on plasma samples collected at enrolment and discharge. This work will be done in collaboration with laboratories at Duke-NUS in Singapore.
8. With separate consent, cells from participant plasma samples are separated and stored for assessment of host genetic factors associated with disease severity using exome sequencing at The Wellcome Trust Sanger Institute, UK.

#### Adverse Events

Any unfavorable or unexpected sign, including an abnormal laboratory finding, which develops or worsens after commencing the study drug infusion, is documented as an Adverse Event (AE). All clinical AEs are recorded on an AE form, but laboratory abnormalities are only entered on an AE Form if they are accompanied by clinical symptoms, lead to withdrawal of the study drug or modification of the study drug dose, or require a change in concomitant therapy.

Serious Adverse Event (SAEs) are defined as those that result in death, are life threatening at the time, require prolongation of existing hospitalization (>14 days, Additional file 1: Appendix 4), or lead to persistent or significant disability/incapacity.

All SAEs are reported to a) OUCRU as the Sponsor and b) the IRB at the relevant site (HTD or ND1) as soon as possible. A report of any SAE that is life threatening or that results in death is also sent to the office of the Vietnamese MoH Research Ethics Committee. These reports are sent within 7 days of knowledge of the event and include details of the event and the recommendation from the site IRB. If all information is not available upon the initial report, a complete report is sent within 15 days of knowledge of the event. All other SAEs are reported to the MoH Research Ethics Committee, including the recommendation of the site ethical committee, within 15 days of knowledge of the event.

### Data management

#### Source data

Source documents are original documents, data, and records from which participants’ study data are obtained. These include, but are not limited to, hospital records (from which medical history and previous and concurrent medication may be summarized into the CRF), digital or printed laboratory and pharmacy records, MRI data files and digital or printed output from monitoring devices. CRF entries are considered source data if the CRF is the site of the original recording (that is, no other written or electronic records of the data exist). In this study, the CRF is used as the source document for some clinical data points. Whenever possible, clinical laboratory data (hematology/biochemistry, etc.) are downloaded directly from the analyzers into the study database.

All documents are stored safely in secure, confidential locations. In order to maintain patient confidentiality, only the signed informed consent form, the patient master log, and the trial drug documents are labeled with the patient’s name or identifying information. All other study documents are identified by the subject number and initials only. Data recorded on paper are entered on site into the database in accordance with standard operating procedures and monitored for accuracy.

Where possible, patients admitted to the HDU/PICU who might potentially be eligible for the study are monitored on systems with the facility for subsequent automatic download of hemodynamic parameters. If the patient does enter the trial and gives appropriate consent, then these data for up to 2 h prior to enrollment are downloaded together with the information for the 72 h after enrollment.

#### Quality control and quality assurance procedures

The study will be conducted in accordance with the current approved protocol, Viet Nam Guidelines for Good Clinical Practice, and study standard operating procedures. The OUCRU Clinical Trials Unit will be engaged in assuring good governance, regulatory compliance, and QA/QC of study execution. Any changes to study documents will be approved by the responsible ethics committee as required and communicated to all study staff by the Principal Investigator before implementation.

#### Trial monitoring

The trial is being monitored according to procedures defined by the OUCRU Research Governance Team to ensure that the rights and safety of patients are protected, and that data are generated, documented, and reported in compliance with the protocol, standard operating procedures, and the appropriate regulatory requirements. Periodic onsite monitoring will take place to verify source documents for study data quality and regulatory compliance according to a risk-based monitoring plan.

#### Data sharing

The final data set will be anonymized and made available through governed sharing mechanisms to external investigators for use in secondary analyses.

### Statistical considerations

#### Sample size

During 2011, approximately one-third of patients with Grade 3 HFMD managed at HTD (48/140, 34 %) and CH1 (48/148, 32 %) required milrinone for ANS dysregulation with hypertension, and in 9/48 (19 %) of the HTD cases and 10/48 (21 %) of the CH1 cases, milrinone, when used alone, failed to control the hypertension (personal communication, Dr. Phan Tu Qui). Following introduction of new Vietnamese MoH management guidelines to commence intra-arterial BP monitoring early (at the first sign of autonomic disturbance), among 16 children managed in this way at HTD over a 2-month period, 12/16 patients developed Stage 1 hypertension, and seven of these 12 cases developed Stage 2 hypertension and were treated with milrinone. Based on this observation (that is, that 7/12 children with Stage 1 hypertension progressed), for the sample size calculation for this study, we estimated a progression rate of 50 % in the control arm.

With respect to the hypothesized treatment effect, little direct information exists for the actual scenario we plan to investigate, that is, the influence of MgSO_4_ commenced at Stage 1 hypertension on subsequent control of SBP and progression to severe disease. In the case series described earlier, when MgSO_4_ was added to the treatment regimen of patients with poorly controlled hypertension, despite high-dose milrinone, in all cases, the SBP reduced within 30 to 60 min and thereafter remained stable on a continuous MgSO_4_ infusion. In the tetanus study mentioned above (in which the study drug was given to patients with severe tetanus requiring a tracheostomy), the requirement for additional therapy to treat ANS dysfunction was reduced from 14/97 (14 %) in the placebo group to 3/97 (3 %) in the MgSO_4_ group, although the need for assisted ventilation was similar in the two groups. Thus, indirect evidence suggests that the effect size of the proposed intervention may be large; we therefore estimate that use of MgSO_4_ could reduce the risk of progression by > 50 %.

Based on 1:1 randomization, an anticipated relative reduction in the risk of progression of 50 % (from 50 % in the control arm to 25 % in MgSO_4_ recipients), 90 % power, and a two-sided 5 % significance level, 85 patients per treatment group are required. To allow for some violations of our assumptions and losses to follow-up, we plan to recruit 190 patients (95 patients per treatment arm) into the study.

#### Statistical analysis

All planned statistical analyses will be pre-defined in a detailed statistical analysis plan, which will be finalized prior to unblinding the trial. The primary analysis population will include all randomized patients who commenced the loading dose infusion of the study drug, and analysis will be according to the randomized treatment arm (intention-to-treat). A per protocol analysis will also be performed, including all subjects who complete the full treatment schedule for 72 h or if death or one of the pre-specified criteria for stopping the study drug occurs within 72 h. An additional analysis will also be performed in the subgroup of patients with confirmed EV71 infection. The primary endpoint of disease progression will be compared between the two treatment arms based on logistic regression with the treatment assignment as the only covariate. As disease progression is evaluated in a short time frame, that is, within 72 h of study drug initiation, we expect to lose only a minimal number of patients to follow-up before that time point and will analyze these patients according to their last recorded disease status. We will also perform regression analysis, including a number of baseline covariates – age, sex, BMI, day of illness at study entry, study site, and baseline severity assessed in terms of an internationally recognized score (PRISM III).

Predefined secondary endpoints and exploratory investigations will be compared between the two treatment arms based on linear regression for continuous endpoints, logistic regression for binary endpoints, and Cox regression for time-to-event endpoints. For laboratory markers, analyses will be adjusted for the pre-dose value of the respective marker. For host genetics analyses in this sample, putative severity alleles will be detected by filtering and comparison with reference data from control groups including the 1000 Genomes Project. For alleles with significant enrichment among severe cases, validation will be attempted using samples from other cohorts with severe HFMD.

The values for CO, SVR, catecholamine, and cytokine measurements will also be correlated with clinical information using descriptive statistical methods. Clinical information including temperature, skin perfusion, capillary refill time, respiratory rate, and urine output will be summarized for defined time intervals (6 hourly), and relations will be assessed with the cardiovascular and biochemical markers measured during the same time interval.

All AEs and SAEs will be recorded in the patient CRF, and reported to the relevant authorities as indicated previously. In addition, for analysis of safety endpoints, the intensity of clinical and laboratory AEs are graded on a five-point scale adapted from the NIH guidelines (CTCAE version 4.03) [29] to ensure that the cutoffs used are appropriate to the age of the population being studied (Additional file 1: Appendix 10).

### Ethical considerations

#### Ethical review

The study is being conducted in compliance with the current revision of the Declaration of Helsinki (Seoul 2008) and the terms of approval of all supervising Ethical Committees. The study protocol and its associated documents have been reviewed and approved by the following committees: The Vietnam Ministry of Health’s Evaluation Committee on Ethics in Biomedical Research, The Ethics Committee of the Hospital for Tropical Diseases, The Science and Ethics Committee in Biomedical Research of Childrens Hospital 1, and the Oxford Tropical Research Ethics Committee (OxTREC, University of Oxford). The study is managed by the OUCRU Clinical Trials Unit in compliance with the ICH Guidelines of Good Clinical Practice, the Vietnamese Ministry of Health Guidelines of Good Clinical Practice and the relevant institutional and regulatory requirements. The study is sponsored by the University of Oxford and appropriate insurance backed indemnity is in place with respect to the University’s role as research sponsor.

#### Informed consent (Additional file 1: Appendix 7)

The study staff members discuss the study with the accompanying parent/guardian, describing the purpose, procedures, possible risks/benefits, the rights and responsibilities of participants, and alternatives to enrollment, and the family are invited to ask questions. Those who agree to participate are asked to sign/date an Informed Consent Form, with a second copy to keep.

As indicated above, because HFMD may progress rapidly, the parents/guardians of potentially eligible patients are given study information shortly after admission, so later if the child fulfils the necessary enrolment criteria, the staff can discuss the study again, allowing the family at least 1 to 2 h to decide. Families of children presenting with Stage 2 hypertension are given up to 30 min to decide, but since insertion of the access lines necessary for standard care takes at least 30 min, this process does not result in delay. All families are given the opportunity to review the study information on the PIS with a study doctor at least once more 12 to 36 h after enrollment.

#### Confidentiality / participant protection

Personal information is recorded for the purpose of consent and to enable follow up of treatment outcomes. Access to identifying information is restricted to site-specific study staff and the clinical care team, and only anonymized data are entered into the study database. All other study documents are identified by the subject number and patient initials only. All documents are stored safely in secure, confidential locations. Data recorded on paper are entered on site into the database in accordance with standard operating procedures and are monitored for accuracy.

#### Patient benefits & compensation

For all trial participants, the medical costs associated with diagnosis and treatment of HFMD are covered from the time that the parent/guardian agrees to allow their child to enroll in the study until discharge but do not include the medical costs related to previous treatment decisions made before enrollment. All trial-related procedures are covered by the study, and patients are reimbursed the cost of local transport to attend for the follow-up visit according to OUCRU compensation policy. This policy reflects the actual cost of transportation plus modest compensation for time lost from work.

#### Withdrawal of participants from the study

Dedicated study staff members are employed to support parents/guardians to meet study visits and ensure that all trial outcome data are collected. Parents/guardians of the study participants may voluntarily withdraw from the study for any reason. If this occurs, the child will be managed in accordance with standard clinical care guidelines. With the agreement of the parent/guardian, a clinical assessment will be performed and recorded at the time of withdrawal, and the reason for withdrawal will also be recorded in the CRF.

#### Communication/dissemination policies

The study protocol and materials will be made available via publication. The results of the study will be reported in international medical journals with authorship determined according to the guidelines of the International Committee of Medical Journal Editors. Participants will not be individually informed of the results. The overall study findings will be published in both English and Vietnamese and reported to the Department of Health of Ho Chi Minh City. The researchers will also convey the research results to the community in Vietnamese through the OUCRU website and through talks and articles written and published in the mainstream media.

#### Safety reviews

An independent Data and Safety Monitoring Board (DSMB) has been established, consisting of qualified individuals with the necessary knowledge of local treatment guidelines, clinical trials, and statistics, and operates in accordance with a written DSMB charter. The DSMB reviewed and approved the conduct of the study after enrollment of the first five, and subsequently, the first 20 patients. In due course the DSMB will perform a formal safety review after 30 patients have been enrolled (phase 1), including unblinded summary tables of SAEs, AEs, or event reports submitted to the DSMB, plus an analysis of overall clinical outcome based on the interim analysis plan. If no safety concerns are identified, recruitment will expand to include CH1 (phase 2). A further DSMB review will take place after 100 patients have enrolled in the study, with additional safety reviews performed annually or at the discretion of the DSMB based on available data and ongoing reporting. Unless the benefit of MgSO_4_ is shown “beyond reasonable doubt,” no formal stopping rule for efficacy is foreseen; the Haybittle-Peto boundary, requiring *P* < 0.001 at interim analysis to consider stopping for efficacy, will be used as a guide.

## Discussion

Although HFMD has become a major contributor to childhood morbidity and mortality in Vietnam, current management strategies rely on guidelines that are based primarily on expert opinion plus two small clinical intervention studies, one of which used retrospective controls. The efficacy of milrinone, an expensive drug with a significant toxicity profile, for management of ANS dysregulation in children with HFMD and brainstem involvement is unclear, and MgSO_4_, an alternative therapeutic agent that is cheap, safe, and easily available may be more effective.

The current Vietnamese MoH guidelines specify that milrinone should be given to children with HFMD and Stage 2 hypertension due to ANS dysregulation. Although a direct comparison of MgSO_4_ with milrinone might have been illuminating, we were constrained to ensure that none of these very sick children be denied the possible benefit of milrinone. Thus, enrollment to this study was designed to be early, when hypertension is first identified (at Stage 1), so that in the event of treatment failure, the MoH treatment guidelines can still be applied. If MgSO_4_ is shown to be effective in controlling ANS dysregulation at this early stage, and in preventing deterioration/development of severe complications, this would be important to pediatric care throughout the Asian region. Additionally, it might be possible later to consider a direct comparison between milrinone and MgSO_4_ in HFMD cases with established severe hypertension (Stage 2).

Sample size estimates for a study of this nature, where reliable baseline outcome data are lacking, are always difficult to calculate. We used indirect evidence from use of MgSO_4_ in other conditions, together with the data from the small, unblinded pilot study in severe HFMD cases that failed to respond to maximum dose milrinone, to estimate the effect size of the proposed intervention. We acknowledge that the proposed effect size is large, but it does appear reasonable based on this evidence. Moreover, given the epidemic situation in Asia, we chose not to delay starting the study further by doing a formal pilot study.

Vietnamese guidelines for the management of HFMD are regularly reviewed, and the MoH Guidelines Committee strongly supports the idea for this study. If the results are positive, it is likely that use of MgSO_4_ would be incorporated promptly into the local guidelines, with the potential for rapid adoption by neighboring countries experiencing similar severe HFMD outbreaks.

### Trial status

To date, 21 cases have been enrolled (July 2015), all of whom survived and were discharged home. Long-term follow-up is in progress for these cases. Interim reports on the first five, and subsequently the first 20, cases were reviewed by the DSMB, which approved the conduct of the study and that no toxicity or danger signals were apparent in the data collected so far. The Vietnamese MoH also audited the trial in April 2015 (as part of a planned review of all intervention studies currently being performed at OUCRU) and approved the study to proceed without modifications. A formal interim analysis for safety is planned after the first 30 patients have been enrolled.

This protocol is based on the approved version 1.6 dated 14 SEP 13, with additions according to publication requirements.

## Additional file

Additional file 1:
**Appendices: Please see details in separate appendices file.** **Appendix 1**: Vietnamese MoH HFMD classification and management guidelines. **Appendix 2**: Suggested indications for specific interventions following Vietnamese MoH guidelines for HFMD. **Appendix 3**: Definitions for hypertension in the study population. **Appendix 4**: Additional study definitions. **Appendix 5**: Magnesium sulfate background information. **Appendix 6**: Rescue treatment guidelines. **Appendix 7**: Patient information sheet & informed consent form. **Appendix 8**: Study schedule for all planned investigations. **Appendix 9**: Laboratory schedule (clinical and research investigations). **Appendix 10**: Modified Adverse Events Grading for HFMD trial. (DOCX 193 kb)

## Abbreviations

AEs: adverse events
ANS: autonomic nervous system
BMI: Body Mass index
BP: blood pressure
CA16: coxsackievirus A16
CH1: Children’s Hospital Number One
CKMB: creatine kinase MB
CO: cardiac output
CRF: case report form
CSF: cerebrospinal fluid
CTCAE: Common Terminology Criteria for Adverse Events
DSMB: data and safety monitoring board
EV71: enterovirus 71
HDU: High Dependency Unit
HFMD: hand, foot, and mouth disease
HTD: Hospital for Tropical Diseases
ICH: The International Conference for Harmonisation of Technical Requirements for Registration of Pharmaceuticals forHuman Use
IL-10: interleukin-10
IL-6: interleukin-6
IL1: interleukin-1
IL13: interleukin-13
INFγ: interferon gamma
IVIG: intravenous immunoglobulin
LiDCO: Lithium Dilution Cardiac Output
MgSO4: magnesium sulfate
MoH: Ministry of Health
NUS: National University of Singapore
OUCRU: Oxford University Clinical Research Unit
PCR: polymerase chain reaction
PICU: Pediatric Intensive Care Unit
PIS: patient information sheet
PRISM III: The Pediatric Risk of Mortality III
SAEs: serious adverse events
SBP: systolic blood pressure
SVR: systemic vascular resistance
TNF-α: tumor necrosis factor-alpha
UK: United Kingdom
