# Supplementary material for: Intravenous magnesium sulfate for the management of severe hand, foot, and mouth disease with autonomic nervous system dysregulation in Vietnamese children: study protocol for a randomized controlled trial
Source: Trials. 2016 Feb 19;17:98. doi: 10.1186/s13063-016-1215-6 (PMC4759733; doi:10.1186/s13063-016-1215-6)
Supplement: Additional file 1: — Appendices: Please see details in separate appendices file. Appendix 1: Vietnamese MoH HFMD classification and management guidelines. Appendix 2: Suggested indications for specific interventions following Vietnamese MoH guidelines for HFMD. Appendix 3: Definitions for hypertension in the study population. Appendix 4: Additional study definitions. Appendix 5: Magnesium sulfate background information. Appendix 6: Rescue treatment guidelines. Appendix 7: Patient information sheet & informed consent form. Appendix 8: Study schedule for all planned investigations. Appendix 9: Laboratory schedule (clinical and research investigations). Appendix 10: Modified Adverse Events Grading for HFMD trial. (DOCX 193 kb) [file 13063_2016_1215_MOESM1_ESM.docx]

**APPENDIX 1: VIETNAMESE MOH HFMD CLASSIFICATION AND MANAGEMENT GUIDELINES [**[**1**](#_ENREF_1)**]**

| Classification | Signs/symptoms | Suggested Management |
| --- | --- | --- |
| Grade 1 | Oral ulcers and/or vesicular rash on the hands, feet, and/or the buttocks | Outpatient care, with advice sheet for family  Careful observation for warning signs |
| Grade 2a | Grade 1 AND   - Myoclonic jerk, but only observed by the family (not witnessed by medical staff) - Lethargy, agitation/irritability - Fever ≥ 39°C or ≥ 48 hours - Vomiting | Hospitalization   - Oral phenobarbitone |
| Grade 2b  - Group 1 | Grade 1 AND   - Myoclonic jerks witnessed by medical staff or by the family (≥ 2 jerks /30 minutes or 1 jerk and stupor) - Resting pulse rate > 130/min but <150/min (adjusted for fever*) | Admit to HDU/PCIU   - IV Phenobarbitone - Antipyretics - Vital Signs Monitoring: every 1-3 hours for > 6 hrs |
| Grade 2b  - Group2 | Grade 1 AND myoclonic jerks accompanied by one of the following findings:   - Continuous limb tremor, limb weakness or paralysis, or drowsiness (provided no hypoglycemia) - Resting pulse rate > 150 /min (adjusted for fever*) - Fever ≥ 39^0^5C (rectal T^0^) and unresponsive to antipyretics over 4 hours | Admit to HDU/PICU   - Give oxygen - IV Phenobarbitone - Antipyretics - Start IVIG – 2g/kg in two divided doses - Check: FBC, CRP, blood sugar, and consider lumbar puncture - Vital signs monitoring: every 1-3 hours for > 6hrs - Consider invasive BP monitoring |
| Grade 3 | Serious complications in CNS or cardiopulmonary systems:   - Pulse >170 /min* - Profuse sweating - Hypertension (SBP > 95^th^ percentile for age, approximately) - Respiratory abnormalities   - Tachypnea   - Labored breathing - Seizures - Coma (Glasgow coma score < 10) | Admit to HDU/PICU   - Give oxygen - Consider need for ventilation - IV Phenobarbitone - IVIG (as for Grade 2b) - Milrinone if systolic blood pressure > 99^th^ percentile for age + 5 mmHg (approximately) - Dobutamine if HR > 170 beats/min - Consider additional fever control measures - Invasive blood pressure monitoring - Check: FBC, CRP, blood sugar, and consider lumbar Puncture - Vital signs monitoring: every 30-60 mins for ≥ 6 hrs |
| Grade 4 | Severe complications:   - Acute pulmonary edema - Cardiac collapse - SpO2 < 92% with cannula oxygen 6 litres/min) - Respiratory arrest / gasping respirations | Admit to HDU/PICU   - Intubation and ventilation - Dobutamine - IV Phenobarbitone - Fluid challenge - Antipyretics - Access CVP - Invasive blood pressure monitoring - Vital signs monitoring: every 15-30 mins for ≥6 hrs |

Note: *: heart rate is adjusted down by 10 beats/min for each 1 degree Celsius above 37^o^C

**APPENDIX 2: SUGGESTED INDICATIONS FOR SPECIFIC INTERVENTIONS FOLLOWING VIETNAMESE MOH GUIDELINES FOR HFMD**

**Ventilation criteria:**

- If a patient continues to display any of the following criteria despite oxygenation via nasal cannula and cardiac support with inotropic drugs for more than 60 minutes.
  - Labored breathing
  - Tachypnea with resting respiratory rate > 70 / minute without fever
  - Hypoxemia and/or fluctuating SpO2
  - Poor tissue perfusion and persistent resting HR > 180 beats/minute without fever
- Or
  - Decorticate or decerebrate rigidity
  - Coma (GCS < 10 )

**Hemofiltration indication criteria**:

- Acute renal failure and one of the following:
  - Severe respiratory distress: Ventilation with FiO_2_ > 60%, inspiratory pressure > 25 cmH_2_O and PEEP > 10 cm H_2_O
  - Unstable hemodynamic status despite intensive resuscitation for 3 hours
  - Coagulopathy (INR> 1,5 )
  - Acute hepatic failure
  - GCS < 10

Or

- Ventilated patients with one or more of the following
  - Coma and refractory fever
  - Coma and refractory shock (shock status not improved after two hours of intensive resuscitation)
  - Heart failure or a positive Troponin I

**APPENDIX 3: DEFINITIONS FOR HYPERTENSION IN THE STUDY POPULATION**

## Hypertension in Children

## The following definitions for hypertension in children are taken from the 2004 (US) national high blood pressure education program working group (NHBPEP) [[2](#_ENREF_2)] All percentiles refer to the relevant value for age, gender and length.

### Normal BP — both systolic and diastolic BP <90th percentile

### Prehypertension — systolic and/or diastolic BP ≥ 90th percentile but <95th percentile or if BP exceeds 120/80 mmHg (even if <90th percentile).

### Hypertension:

#### Stage 1 HTN — systolic and/or diastolic BP between the 95th percentile and 5 mmHg above the 99th percentile.

#### Stage 2 HTN — systolic and/or diastolic BP > 99th percentile plus 5 mmHg.

- - Hypertensive emergency: A severe symptomatic elevation in BP (> 30% compared to baseline blood pressure) WITH evidence of acute target organ damage defines a hypertensive emergency
    - Brain (seizures, increased intracranial pressure)
    - Kidneys (renal insufficiency)
    - Eyes (papilledema, retinal hemorrhages, exudates)
    - Heart (heart failure)

**Hypertension in infants (6-12 months) [**[**3**](#_ENREF_3)**]:**

Because there are no normative data that describe 95th percentile BP values for infants less than one year of age, and the blood pressure is almost unchanged in infants from 6 months to 12 months, the following thresholds will be used to identify hypertension in infants:

- - An invasive blood pressure measurement of >100/60 will be taken as indicating Stage 1 hypertension. (An oscillometric awake BP of > 100/60 is accepted as the level at which follow-up BP monitoring and an evaluation for an underlying cause for elevated BP is usually recommended).
  - An invasive blood pressure measurement that is persistently **≥** 110/65 will be considered as Stage 2 hypertension. (Treatment is generally initiated for BP persistently **≥** 110/65, or sooner if left ventricular hypertrophy is present).

**BP Levels for Girls by Age and Height Percentile**

| Age, y | BP Percentile |  |  | SBP, mm Hg | | |  |  | DBP, mm Hg | | | | |  | |
| --- | --- | --- | --- | --- | --- | --- | --- | --- | --- | --- | --- | --- | --- | --- | --- |
|  |  |  |  | Percentile of Height | | |  |  | Percentile of Height | | | | |  |  |
|  |  | 5th | 10th | 25th | 50th | 75th | 90th | 95th | 5th | 10th | 25th | 50th | 75th | 90th | 95th |
| 1 | 50th | 83 | 84 | 85 | 86 | 88 | 89 | 90 | 38 | 39 | 39 | 40 | 41 | 41 | 42 |
|  | 90th | 97 | 97 | 98 | 100 | 101 | 102 | 103 | 52 | 53 | 53 | 54 | 55 | 55 | 56 |
|  | 95th | 100 | 101 | 102 | 104 | 105 | 106 | 107 | 56 | 57 | 57 | 58 | 59 | 59 | 60 |
|  | 99th | 108 | 108 | 109 | 111 | 112 | 113 | 114 | 64 | 64 | 65 | 65 | 66 | 67 | 67 |
| 2 | 50th | 85 | 85 | 87 | 88 | 89 | 91 | 91 | 43 | 44 | 44 | 45 | 46 | 46 | 47 |
|  | 90th | 98 | 99 | 100 | 101 | 103 | 104 | 105 | 57 | 58 | 58 | 59 | 60 | 61 | 61 |
|  | 95th | 102 | 103 | 104 | 105 | 107 | 108 | 109 | 61 | 62 | 62 | 63 | 64 | 65 | 65 |
|  | 99th | 109 | 110 | 111 | 112 | 114 | 115 | 116 | 69 | 69 | 70 | 70 | 71 | 72 | 72 |
| 3 | 50th | 86 | 87 | 88 | 89 | 91 | 92 | 93 | 47 | 48 | 48 | 49 | 50 | 50 | 51 |
|  | 90th | 100 | 100 | 102 | 103 | 104 | 106 | 106 | 61 | 62 | 62 | 63 | 64 | 64 | 65 |
|  | 95th | 104 | 104 | 105 | 107 | 108 | 109 | 110 | 65 | 66 | 66 | 67 | 68 | 68 | 69 |
|  | 99th | 111 | 111 | 113 | 114 | 115 | 116 | 117 | 73 | 73 | 74 | 74 | 75 | 76 | 76 |
| 4 | 50th | 88 | 88 | 90 | 91 | 92 | 94 | 94 | 50 | 50 | 51 | 52 | 52 | 53 | 54 |
|  | 90th | 101 | 102 | 103 | 104 | 106 | 107 | 108 | 64 | 64 | 65 | 66 | 67 | 67 | 68 |
|  | 95th | 105 | 106 | 107 | 108 | 110 | 111 | 112 | 68 | 68 | 69 | 70 | 71 | 71 | 72 |
|  | 99th | 112 | 113 | 114 | 115 | 117 | 118 | 119 | 76 | 76 | 76 | 77 | 78 | 79 | 79 |
| 5 | 50th | 89 | 90 | 91 | 93 | 94 | 95 | 96 | 52 | 53 | 53 | 54 | 55 | 55 | 56 |
|  | 90th | 103 | 103 | 105 | 106 | 107 | 109 | 109 | 66 | 67 | 67 | 68 | 69 | 69 | 70 |
|  | 95th | 107 | 107 | 108 | 110 | 111 | 112 | 113 | 70 | 71 | 71 | 72 | 73 | 73 | 74 |
|  | 99th | 114 | 114 | 116 | 117 | 118 | 120 | 120 | 78 | 78 | 79 | 79 | 80 | 81 | 81 |
| 6 | 50th | 91 | 92 | 93 | 94 | 96 | 97 | 98 | 54 | 54 | 55 | 56 | 56 | 57 | 58 |
|  | 90th | 104 | 105 | 106 | 108 | 109 | 110 | 111 | 68 | 68 | 69 | 70 | 70 | 71 | 72 |
|  | 95th | 108 | 109 | 110 | 111 | 113 | 114 | 115 | 72 | 72 | 73 | 74 | 74 | 75 | 76 |
|  | 99th | 115 | 116 | 117 | 119 | 120 | 121 | 122 | 80 | 80 | 80 | 81 | 82 | 83 | 83 |
| 7 | 50th | 93 | 93 | 95 | 96 | 97 | 99 | 99 | 55 | 56 | 56 | 57 | 58 | 58 | 59 |
|  | 90th | 106 | 107 | 108 | 109 | 111 | 112 | 113 | 69 | 70 | 70 | 71 | 72 | 72 | 73 |
|  | 95th | 110 | 111 | 112 | 113 | 115 | 116 | 116 | 73 | 74 | 74 | 75 | 76 | 76 | 77 |
|  | 99th | 117 | 118 | 119 | 120 | 122 | 123 | 124 | 81 | 81 | 82 | 82 | 83 | 84 | 84 |
| 8 | 50th | 95 | 95 | 96 | 98 | 99 | 100 | 101 | 57 | 57 | 57 | 58 | 59 | 60 | 60 |
|  | 90th | 108 | 109 | 110 | 111 | 113 | 114 | 114 | 71 | 71 | 71 | 72 | 73 | 74 | 74 |
|  | 95th | 112 | 112 | 114 | 115 | 116 | 118 | 118 | 75 | 75 | 75 | 76 | 77 | 78 | 78 |
|  | 99th | 119 | 120 | 121 | 122 | 123 | 125 | 125 | 82 | 82 | 83 | 83 | 84 | 85 | 86 |
| 9 | 50th | 96 | 97 | 98 | 100 | 101 | 102 | 103 | 58 | 58 | 58 | 59 | 60 | 61 | 61 |
|  | 90th | 110 | 110 | 112 | 113 | 114 | 116 | 116 | 72 | 72 | 72 | 73 | 74 | 75 | 75 |
|  | 95th | 114 | 114 | 115 | 117 | 118 | 119 | 120 | 76 | 76 | 76 | 77 | 78 | 79 | 79 |
|  | 99th | 121 | 121 | 123 | 124 | 125 | 127 | 127 | 83 | 83 | 84 | 84 | 85 | 86 | 87 |
| 10 | 50th | 98 | 99 | 100 | 102 | 103 | 104 | 105 | 59 | 59 | 59 | 60 | 61 | 62 | 62 |
|  | 90th | 112 | 112 | 114 | 115 | 116 | 118 | 118 | 73 | 73 | 73 | 74 | 75 | 76 | 76 |
|  | 95th | 116 | 116 | 117 | 119 | 120 | 121 | 122 | 77 | 77 | 77 | 78 | 79 | 80 | 80 |
|  | 99th | 123 | 123 | 125 | 126 | 127 | 129 | 129 | 84 | 84 | 85 | 86 | 86 | 87 | 88 |
| 11 | 50th | 100 | 101 | 102 | 103 | 105 | 106 | 107 | 60 | 60 | 60 | 61 | 62 | 63 | 63 |
|  | 90th | 114 | 114 | 116 | 117 | 118 | 119 | 120 | 74 | 74 | 74 | 75 | 76 | 77 | 77 |
|  | 95th | 118 | 118 | 119 | 121 | 122 | 123 | 124 | 78 | 78 | 78 | 79 | 80 | 81 | 81 |
|  | 99th | 125 | 125 | 126 | 128 | 129 | 130 | 131 | 85 | 85 | 86 | 87 | 87 | 88 | 89 |
| 12 | 50th | 102 | 103 | 104 | 105 | 107 | 108 | 109 | 61 | 61 | 61 | 62 | 63 | 64 | 64 |
|  | 90th | 116 | 116 | 117 | 119 | 120 | 121 | 122 | 75 | 75 | 75 | 76 | 77 | 78 | 78 |
|  | 95th | 119 | 120 | 121 | 123 | 124 | 125 | 126 | 79 | 79 | 79 | 80 | 81 | 82 | 82 |
|  | 99th | 127 | 127 | 128 | 130 | 131 | 132 | 133 | 86 | 86 | 87 | 88 | 88 | 89 | 90 |
| 13 | 50th | 104 | 105 | 106 | 107 | 109 | 110 | 110 | 62 | 62 | 62 | 63 | 64 | 65 | 65 |
|  | 90th | 117 | 118 | 119 | 121 | 122 | 123 | 124 | 76 | 76 | 76 | 77 | 78 | 79 | 79 |
|  | 95th | 121 | 122 | 123 | 124 | 126 | 127 | 128 | 80 | 80 | 80 | 81 | 82 | 83 | 83 |
|  | 99th | 128 | 129 | 130 | 132 | 133 | 134 | 135 | 87 | 87 | 88 | 89 | 89 | 90 | 91 |
| 14 | 50th | 106 | 106 | 107 | 109 | 110 | 111 | 112 | 63 | 63 | 63 | 64 | 65 | 66 | 66 |
|  | 90th | 119 | 120 | 121 | 122 | 124 | 125 | 125 | 77 | 77 | 77 | 78 | 79 | 80 | 80 |
|  | 95th | 123 | 123 | 125 | 126 | 127 | 129 | 129 | 81 | 81 | 81 | 82 | 83 | 84 | 84 |
|  | 99th | 130 | 131 | 132 | 133 | 135 | 136 | 136 | 88 | 88 | 89 | 90 | 90 | 91 | 92 |
| 15 | 50th | 107 | 108 | 109 | 110 | 111 | 113 | 113 | 64 | 64 | 64 | 65 | 66 | 67 | 67 |
|  | 90th | 120 | 121 | 122 | 123 | 125 | 126 | 127 | 78 | 78 | 78 | 79 | 80 | 81 | 81 |
|  | 95th | 124 | 125 | 126 | 127 | 129 | 130 | 131 | 82 | 82 | 82 | 83 | 84 | 85 | 85 |
|  | 99th | 131 | 132 | 133 | 134 | 136 | 137 | 138 | 89 | 89 | 90 | 91 | 91 | 92 | 93 |
| 16 | 50th | 108 | 108 | 110 | 111 | 112 | 114 | 114 | 64 | 64 | 65 | 66 | 66 | 67 | 68 |
|  | 90th | 121 | 122 | 123 | 124 | 126 | 127 | 128 | 78 | 78 | 79 | 80 | 81 | 81 | 82 |
|  | 95th | 125 | 126 | 127 | 128 | 130 | 131 | 132 | 82 | 82 | 83 | 84 | 85 | 85 | 86 |
|  | 99th | 132 | 133 | 134 | 135 | 137 | 138 | 139 | 90 | 90 | 90 | 91 | 92 | 93 | 93 |
| 17 | 50th | 108 | 109 | 110 | 111 | 113 | 114 | 115 | 64 | 65 | 65 | 66 | 67 | 67 | 68 |
|  | 90th | 122 | 122 | 123 | 125 | 126 | 127 | 128 | 78 | 79 | 79 | 80 | 81 | 81 | 82 |
|  | 95th | 125 | 126 | 127 | 129 | 130 | 131 | 132 | 82 | 83 | 83 | 84 | 85 | 85 | 86 |
|  | 99th | 133 | 133 | 134 | 136 | 137 | 138 | 139 | 90 | 90 | 91 | 91 | 92 | 93 | 93 |

| **BP Levels for Boys by Age and Height Percentile** | | | | | | | | | | | | | | | |
| --- | --- | --- | --- | --- | --- | --- | --- | --- | --- | --- | --- | --- | --- | --- | --- |
| Age, y | BP Percentile |  |  | SBP, mm Hg | | |  |  | DBP, mm Hg | | | | |  | |
|  |  |  |  | Percentile of Height | | |  |  | Percentile of Height | | | | |  |  |
|  |  | 5th | 10th | 25th | 50th | 75th | 90th | 95th | 5th | 10th | 25th | 50th | 75th | 90th | 95th |
| 1 | 50th | 80 | 81 | 83 | 85 | 87 | 88 | 89 | 34 | 35 | 36 | 37 | 38 | 39 | 39 |
|  | 90th | 94 | 95 | 97 | 99 | 100 | 102 | 103 | 49 | 50 | 51 | 52 | 53 | 53 | 54 |
|  | 95th | 98 | 99 | 101 | 103 | 104 | 106 | 106 | 54 | 54 | 55 | 56 | 57 | 58 | 58 |
|  | 99th | 105 | 106 | 108 | 110 | 112 | 113 | 114 | 61 | 62 | 63 | 64 | 65 | 66 | 66 |
| 2 | 50th | 84 | 85 | 87 | 88 | 90 | 92 | 92 | 39 | 40 | 41 | 42 | 43 | 44 | 44 |
|  | 90th | 97 | 99 | 100 | 102 | 104 | 105 | 106 | 54 | 55 | 56 | 57 | 58 | 58 | 59 |
|  | 95th | 101 | 102 | 104 | 106 | 108 | 109 | 110 | 59 | 59 | 60 | 61 | 62 | 63 | 63 |
|  | 99th | 109 | 110 | 111 | 113 | 115 | 117 | 117 | 66 | 67 | 68 | 69 | 70 | 71 | 71 |
| 3 | 50th | 86 | 87 | 89 | 91 | 93 | 94 | 95 | 44 | 44 | 45 | 46 | 47 | 48 | 48 |
|  | 90th | 100 | 101 | 103 | 105 | 107 | 108 | 109 | 59 | 59 | 60 | 61 | 62 | 63 | 63 |
|  | 95th | 104 | 105 | 107 | 109 | 110 | 112 | 113 | 63 | 63 | 64 | 65 | 66 | 67 | 67 |
|  | 99th | 111 | 112 | 114 | 116 | 118 | 119 | 120 | 71 | 71 | 72 | 73 | 74 | 75 | 75 |
| 4 | 50th | 88 | 89 | 91 | 93 | 95 | 96 | 97 | 47 | 48 | 49 | 50 | 51 | 51 | 52 |
|  | 90th | 102 | 103 | 105 | 107 | 109 | 110 | 111 | 62 | 63 | 64 | 65 | 66 | 66 | 67 |
|  | 95th | 106 | 107 | 109 | 111 | 112 | 114 | 115 | 66 | 67 | 68 | 69 | 70 | 71 | 71 |
|  | 99th | 113 | 114 | 116 | 118 | 120 | 121 | 122 | 74 | 75 | 76 | 77 | 78 | 78 | 79 |
| 5 | 50th | 90 | 91 | 93 | 95 | 96 | 98 | 98 | 50 | 51 | 52 | 53 | 54 | 55 | 55 |
|  | 90th | 104 | 105 | 106 | 108 | 110 | 111 | 112 | 65 | 66 | 67 | 68 | 69 | 69 | 70 |
|  | 95th | 108 | 109 | 110 | 112 | 114 | 115 | 116 | 69 | 70 | 71 | 72 | 73 | 74 | 74 |
|  | 99th | 115 | 116 | 118 | 120 | 121 | 123 | 123 | 77 | 78 | 79 | 80 | 81 | 81 | 82 |
| 6 | 50th | 91 | 92 | 94 | 96 | 98 | 99 | 100 | 53 | 53 | 54 | 55 | 56 | 57 | 57 |
|  | 90th | 105 | 106 | 108 | 110 | 111 | 113 | 113 | 68 | 68 | 69 | 70 | 71 | 72 | 72 |
|  | 95th | 109 | 110 | 112 | 114 | 115 | 117 | 117 | 72 | 72 | 73 | 74 | 75 | 76 | 76 |
|  | 99th | 116 | 117 | 119 | 121 | 123 | 124 | 125 | 80 | 80 | 81 | 82 | 83 | 84 | 84 |
| 7 | 50th | 92 | 94 | 95 | 97 | 99 | 100 | 101 | 55 | 55 | 56 | 57 | 58 | 59 | 59 |
|  | 90th | 106 | 107 | 109 | 111 | 113 | 114 | 115 | 70 | 70 | 71 | 72 | 73 | 74 | 74 |
|  | 95th | 110 | 111 | 113 | 115 | 117 | 118 | 119 | 74 | 74 | 75 | 76 | 77 | 78 | 78 |
|  | 99th | 117 | 118 | 120 | 122 | 124 | 125 | 126 | 82 | 82 | 83 | 84 | 85 | 86 | 86 |
| 8 | 50th | 94 | 95 | 97 | 99 | 100 | 102 | 102 | 56 | 57 | 58 | 59 | 60 | 60 | 61 |
|  | 90th | 107 | 109 | 110 | 112 | 114 | 115 | 116 | 71 | 72 | 72 | 73 | 74 | 75 | 76 |
|  | 95th | 111 | 112 | 114 | 116 | 118 | 119 | 120 | 75 | 76 | 77 | 78 | 79 | 79 | 80 |
|  | 99th | 119 | 120 | 122 | 123 | 125 | 127 | 127 | 83 | 84 | 85 | 86 | 87 | 87 | 88 |
| 9 | 50th | 95 | 96 | 98 | 100 | 102 | 103 | 104 | 57 | 58 | 59 | 60 | 61 | 61 | 62 |
|  | 90th | 109 | 110 | 112 | 114 | 115 | 117 | 118 | 72 | 73 | 74 | 75 | 76 | 76 | 77 |
|  | 95th | 113 | 114 | 116 | 118 | 119 | 121 | 121 | 76 | 77 | 78 | 79 | 80 | 81 | 81 |
|  | 99th | 120 | 121 | 123 | 125 | 127 | 128 | 129 | 84 | 85 | 86 | 87 | 88 | 88 | 89 |
| 10 | 50th | 97 | 98 | 100 | 102 | 103 | 105 | 106 | 58 | 59 | 60 | 61 | 61 | 62 | 63 |
|  | 90th | 111 | 112 | 114 | 115 | 117 | 119 | 119 | 73 | 73 | 74 | 75 | 76 | 77 | 78 |
|  | 95th | 115 | 116 | 117 | 119 | 121 | 122 | 123 | 77 | 78 | 79 | 80 | 81 | 81 | 82 |
|  | 99th | 122 | 123 | 125 | 127 | 128 | 130 | 130 | 85 | 86 | 86 | 88 | 88 | 89 | 90 |
| 11 | 50th | 99 | 100 | 102 | 104 | 105 | 107 | 107 | 59 | 59 | 60 | 61 | 62 | 63 | 63 |
|  | 90th | 113 | 114 | 115 | 117 | 119 | 120 | 121 | 74 | 74 | 75 | 76 | 77 | 78 | 78 |
|  | 95th | 117 | 118 | 119 | 121 | 123 | 124 | 125 | 78 | 78 | 79 | 80 | 81 | 82 | 82 |
|  | 99th | 124 | 125 | 127 | 129 | 130 | 132 | 132 | 86 | 86 | 87 | 88 | 89 | 90 | 90 |
| 12 | 50th | 101 | 102 | 104 | 106 | 108 | 109 | 110 | 59 | 60 | 61 | 62 | 63 | 63 | 64 |
|  | 90th | 115 | 116 | 118 | 120 | 121 | 123 | 123 | 74 | 75 | 75 | 76 | 77 | 78 | 79 |
|  | 95th | 119 | 120 | 122 | 123 | 125 | 127 | 127 | 78 | 79 | 80 | 81 | 82 | 82 | 83 |
|  | 99th | 126 | 127 | 129 | 131 | 133 | 134 | 135 | 86 | 87 | 88 | 89 | 90 | 90 | 91 |
| 13 | 50th | 104 | 105 | 106 | 108 | 110 | 111 | 112 | 60 | 60 | 61 | 62 | 63 | 64 | 64 |
|  | 90th | 117 | 118 | 120 | 122 | 124 | 125 | 126 | 75 | 75 | 76 | 77 | 78 | 79 | 79 |
|  | 95th | 121 | 122 | 124 | 126 | 128 | 129 | 130 | 79 | 79 | 80 | 81 | 82 | 83 | 83 |
|  | 99th | 128 | 130 | 131 | 133 | 135 | 136 | 137 | 87 | 87 | 88 | 89 | 90 | 91 | 91 |
| 14 | 50th | 106 | 107 | 109 | 111 | 113 | 114 | 115 | 60 | 61 | 62 | 63 | 64 | 65 | 65 |
|  | 90th | 120 | 121 | 123 | 125 | 126 | 128 | 128 | 75 | 76 | 77 | 78 | 79 | 79 | 80 |
|  | 95th | 124 | 125 | 127 | 128 | 130 | 132 | 132 | 80 | 80 | 81 | 82 | 83 | 84 | 84 |
|  | 99th | 131 | 132 | 134 | 136 | 138 | 139 | 140 | 87 | 88 | 89 | 90 | 91 | 92 | 92 |
| 15 | 50th | 109 | 110 | 112 | 113 | 115 | 117 | 117 | 61 | 62 | 63 | 64 | 65 | 66 | 66 |
|  | 90th | 122 | 124 | 125 | 127 | 129 | 130 | 131 | 76 | 77 | 78 | 79 | 80 | 80 | 81 |
|  | 95th | 126 | 127 | 129 | 131 | 133 | 134 | 135 | 81 | 81 | 82 | 83 | 84 | 85 | 85 |
|  | 99th | 134 | 135 | 136 | 138 | 140 | 142 | 142 | 88 | 89 | 90 | 91 | 92 | 93 | 93 |
| 16 | 50th | 111 | 112 | 114 | 116 | 118 | 119 | 120 | 63 | 63 | 64 | 65 | 66 | 67 | 67 |
|  | 90th | 125 | 126 | 128 | 130 | 131 | 133 | 134 | 78 | 78 | 79 | 80 | 81 | 82 | 82 |
|  | 95th | 129 | 130 | 132 | 134 | 135 | 137 | 137 | 82 | 83 | 83 | 84 | 85 | 86 | 87 |
|  | 99th | 136 | 137 | 139 | 141 | 143 | 144 | 145 | 90 | 90 | 91 | 92 | 93 | 94 | 94 |
| 17 | 50th | 114 | 115 | 116 | 118 | 120 | 121 | 122 | 65 | 66 | 66 | 67 | 68 | 69 | 70 |
|  | 90th | 127 | 128 | 130 | 132 | 134 | 135 | 136 | 80 | 80 | 81 | 82 | 83 | 84 | 84 |
|  | 95th | 131 | 132 | 134 | 136 | 138 | 139 | 140 | 84 | 85 | 86 | 87 | 87 | 88 | 89 |
|  | 99th | 139 | 140 | 141 | 143 | 145 | 146 | 147 | 92 | 93 | 93 | 94 | 95 | 96 | 97 |

**APPENDIX 4: ADDITIONAL STUDY DEFINITIONS**

## Autonomic dysfunction is defined by WHO as a combination of the following features: heart rate of 150-170 beats/min, systolic blood pressure variability with absolute values higher than the 95^th^ percentile for age, gender and height, profuse sweating, mottled skin, respiratory abnormalities, and hyperglycemia.

**Tachypnea**: respiratory rate sustained above the following levels at rest

- 6 – 12 months ≥ 50
- 13 – 72 months ≥ 40
- 6 – 12 years ≥ 30
- 13 – 15 years ≥ 25

**Refractory Fever:** Core temperature > 40^o^C for at least 4 hours despite antipyretics

**Hyperglycemia**: >150 mg/dl (8.3 mmol/l) on a random test [[4](#_ENREF_4)], or >126 mg/dl (7 mmol/l) on a fasting test (at least 4 hours after feeding).

**Respiratory distress:** if the patient has any one of the following findings[[1](#_ENREF_1)]:

- Tachypnea
- Irregular breathing
- Wheeze
- Stridor
- Cheyne-Stokes breathing
- Gasping respirations
- Apnoeic episodes

**Shock: if patient has one of the following criteria [**[**1**](#_ENREF_1)**]:**

- Pulseless and blood pressure is not measurable
- Systolic Presure < 70 + 2 x age
- Pulse pressure < 25 mmHg

**Cardiac arrhythmia:**

- Sinus tachycardia is not an exclusion criterion, but any other cardiac arrhythmia is, including AV block (Grades I-III) or QT prolonged >0.48 ms

**Acute Renal failure:**

#### Serum creatinine > 2mg/dl (176 μmol/l) or urine output < 1ml/kg/hr for 4 hours or more

**Prolonged hospitalization:** duration of hospitalization > 14 days

**APPENDIX 5: MAGNESIUM SULFATE BACKGROUND INFORMATION**

## *PHARMACOLOGY:*

Magnesium is important as a cofactor in many enzymatic reactions in the body. There are at least 300 enzymes that are dependent upon magnesium for normal functioning. Actions on lipoprotein lipase have been found to be important in reducing serum cholesterol. Magnesium is necessary for the maintaining of serum potassium and calcium levels due to its effect on the renal tubule. In the heart, magnesium acts as a calcium channel blocker. It also activates sodium potassium ATPase in the cell membrane to promote resting polarization and produce arrhythmias. Magnesium prevents premature labor by inhibiting myometrium contractions. In the CNS, magnesium prevents or controls seizures by blocking neuromuscular transmission and decreasing the amount of acetylcholine liberated at the end-plate by the motor nerve impulse. It also has a depressant effect on the CNS.

## *SAFETY IN CLINICAL PRACTICE:*

Although there are few formal research studies in young children magnesium sulfate is generally considered to be safe, even in neonates. A randomised trial that compared magnesium sulfate with placebo for women with pre-eclampsia found that exposure to MgSO_4_ during labor did not effect long term morbidity or mortality among the 827 children involved in the study; neonatal outcomes were similar in the MgSO_4_ and control patients[[5](#_ENREF_5)]. In a Cochrane review of four studies using MgSO_4_ for persistent pulmonary hypertension of the newborn (loading dose of 200 mg/kg MgSO_4_, followed by a continuous infusion of 20 to 150 mg/kg/hour lasting for 72 hours), among 40 term infants treated, no adverse events were reported except for transient bradycardia responsive to dobutamine in one of the studies. [[6](#_ENREF_6)]. Finally, in a meta-analysis of 5 randomised controlled trials assessing use of intravenous MgSO_4_ for treating acute asthma (182 children, doses ranging from 25 mg/kg to 75 mg/kg), the treatment was well tolerated and only minor side effects were reported, such as epigastric or facial warmth, flushing, pain and numbness at infusion site, dry mouth, and malaise [[7](#_ENREF_7)].

## *DOSAGE:*

In a randomized trial in neonates focused on management of pulmonary hypertension the following doses were used: a loading dose of 200 mg/kg MgSO_4_ diluted to 10% in sterile water was given intravenously over 20 minutes, followed by a continuous infusion of 20 to 150 mg/kg/hour lasting 72 hours, aiming to obtain a concentration of serum magnesium from 3.5 to 5.5 mmol/l [[8](#_ENREF_8)]

In a randomized trial in adults with severe tetanus: a loading dose of 2g/hour MgSO_4_ diluted to 10% in sterile water was given intravenously over 30 minutes, followed by a continuous infusion of 40 mg/kg/hour, lasting up to 7 days, aiming for serum magnesium levels of 2 to 4 mmol/l [[9](#_ENREF_9)]

In severe exacerbations of asthma the following regimen is recommended by the Royal Children’s Hospital in Melbourne: A loading dose of 50 mg/kg MgSO_4_ diluted to 10% in sterile water given intravenously over 20 minutes, followed by a continuous infusion of 30 mg/kg/hour lasting 24 -48 hours [[10](#_ENREF_10)]

## *SIDE EFFECTS [*[*11*](#_ENREF_11)*,* [*12*](#_ENREF_12)*]:*

Adverse effects with magnesium therapy are primarily related to the serum magnesium level. The approximate relation between clinical manifestations and the degree of hypermagnesemia can be summarized as follows:

- Plasma Mg concentration > 1.25 mmol/l – impaired peripheral neuromuscular transmission leading to anticonvulsant effects
- Plasma Mg concentration 2 to 3 mmol/L – nausea, flushing, headache, lethargy, drowsiness, and diminished deep tendon reflexes.
- Plasma Mg concentration 3 to 5 mmol/L – somnolence, hypocalcemia, absent deep tendon reflexes, hypotension, bradycardia, and ECG changes.
- Plasma Mg concentration above 5 mmol/L – muscle paralysis, respiratory paralysis, complete heart block, and cardiac arrest. In most cases, respiratory failure precedes cardiac collapse.

Other recognised effects include:

1. Cardiovascular: Although a Mg level > 5 mmol/l may precipitate cardiac arrhythmias, magnesium is also used as a treatment for certain rhythm disturbances – such as irregular/polymorphic VT with normal baseline QT interval

2. Gastrointestinal: Abdominal cramps, diarrhea, gas formation

***DOSING ADJUSTMENT IN RENAL IMPAIRMENT:***

Patients in severe renal failure (creatinine clearance below 10ml/minute) should not receive magnesium due to toxicity as a result of accumulation. Patients with a creatinine clearance of <25 mL/minute receiving magnesium should have serum magnesium levels carefully monitored.

**APPENDIX 6: RESCUE TREATMENT GUIDELINES**

**Cardiac arrhythmia and/or prolonged QT interval:**

When plasma Mg level is high, usually above 5 mmol/l, this may result in prolongation of the QT interval and this could precipitate an arrhythmia. An ECG will be done at baseline and then daily and the QT interval will be measured as a routine. In addition if any arrhythmia is noted on the cardiac monitor the following actions will be taken.

- Assess the patient clinically, perform a full ECG, check plasma Mg and Ca level and discuss the clinical situation with the site PI.
- If the QT interval is above 480 mms, the study drug will be stopped immediately.
- Any other serious cardiac arrhythmia will be managed according to APLS guidelines
- The results of the Mg/Ca levels will go to the independent doctor for review. He/she will discuss the situation with the treating doctor. If the clinician has any clinical concerns the independent doctor will release the blood results so that the treating doctor can take any necessary action immediately. However, if the treating doctor and the site PI consider that the arrhythmia is minor (e.g occasional SVE’s) and there are no other clinical issues of concern, then the blood results will not be released. The independent doctor may recommend adjustments to the study drug infusion according to the blood test results, but sham adjustments will also be made to the placebo arm to maintain blinding.
- However, if the calcium level < 0.9 mmol/l, the independent doctor will inform the treating doctor of this result specifically so that a rescue dose of 0.5ml/kg of calcium gluconate 10% can be given.

**Hypotension***:*

Development of hypotension could be the natural progression of severe disease or a side effect of MgS0_4_, especially if the plasma Mg level is above 3 mmol/l. If hypotension occurs, defined as a drop in systolic blood pressure to below 70mmHg + 2 X the age in years lasting for 15 minutes, the follow actions will be taken:

- Study drug will be stopped immediately
- Assess the patient clinically, check plasma Mg/Ca levels and creatinine, and discuss the clinical situation with the site PI
- Start with a fluid challenge of 5ml/kg of Lactate Ringers or NaCl 0.9% over 15 mins
- Access the central venous pressure (CVP) and titrate the rate of fluid infusion based on CVP measurements and the clinical response.
- Start inotropes or vasopressors (such as dobutamine, noradrenaline or adrenaline), and continue with interventions following the Vietnamese MOH guideline.
- The results of the Mg/Ca levels will go directly to the ward clinicians. If the calcium level is < 0.9 mmol/l, regardless of the plasma Mg level, a rescue dose of 0.5ml/kg of calcium gluconate 10% will be given.
- The plasma Mg/Ca results may unblind the clinical team to the randomization arm and the fact that an urgent level has been reported to the ward doctors will be recorded in the CRF.
- If the creatinine level is increasing and the treating doctor suspects a diagnosis of acute renal failure, there may be a risk of toxicity due to Mg accumulation. Hemofiltration will be commenced according to MOH guidelines regardless of the plasma Mg level.

**Urine output < 1ml/kg/hr:**

If the urine output is < 1ml/kg/ hr over 4 hours the following actions will be taken:

- A bedside ultrasound will be performed to check whether there is any urine in the bladder
- A urethral catheter will be inserted to monitor the urine output closely, and the plasma creatinine will be checked urgently
- If the creatinine has increased to twice the baseline value, the study drug will be stopped and hemofiltration/dialysis will be performed according to the MOH guidelines regardless of the plasma Mg level.
- If the creatinine is in the normal range but the bladder is empty, then the urine output will be monitored closely for 4 hours more. If the urine output is increasing during this time the study drug will be continued, but if there is no improvement the study drug will be stopped and hemofiltration/dialysis will be performed according to the MOH guidelines regardless of the plasma Mg level.
- If the creatinine is in the normal range and there is urine in bladder, the urine output will be monitoring hourly for at least the next 24 hours

**Cardiac arrest:**

In the event of a cardiac arrest the following actions will be taken:

- Stop study drug infusion immediately
- Emergency resuscitation will be started immediately according to APLS guidelines
- Plasma Mg and Ca levels will be checked urgently and the results will be returned directly to the ward clinicians.
- A rescue dose of 0.5ml/kg of calcium gluconate 10% will be given while awaiting the results if other attempts including CPR and conventional resuscitation drugs fail to restore sinus rhythm and effective cardiac output.
- The plasma Mg/Ca results may unblind the clinical team to the randomization arm and the fact that an urgent level has been reported to the ward doctors will be recorded in the CRF.
- Further intensive intervention will be done according to the Vietnamese MOH guidelines for HFMD management and APLS resuscitation guidelines.

**Respiratory muscle weakness:**

If the patient develops new or worsening signs and symptoms of respiratory distress the following actions will be taken:

- Assess the patient clinically including deep tendon reflexes, check the plasma Mg and Ca levels and an arterial blood gas and discuss the clinical situation with the site PI.
- If the patient meets the MOH ventilation criteria as indicated above, (or the treating doctor thinks that the patient needs to be intubated for any reason) the study drug will be stopped immediately,
  - The results of the Mg/Ca levels will go directly to the ward clinicians. If the calcium level is < 0.9 mmol/l, regardless of the plasma Mg level, a rescue dose of 0.5ml/kg of calcium gluconate 10% will be given.
  - The plasma Mg/Ca results may unblind the clinical team to the randomization arm and the fact that an urgent level has been reported to the ward doctors will be recorded in the CRF.
- If the patient does not meet the MOH ventilation criteria and the treating doctor and site PI agree that immediate intervention/respiratory support is not needed the patient will be observed closely for at least 60 minutes.
  - The results of the Mg/Ca levels will go to the independent doctor for review as soon as possible. If the Mg level is above 2.5 mmol/l but below 3 mmol/l, the treating doctor will be informed to reduce the infusion dose as described in the protocol. Similar sham adjustments can also be made to the placebo arm to maintain blinding.
  - If the Mg level is 3 mmol/l or above, the independent doctor will release the result and inform the study doctor to stop the study drug infusion.
  - If the Ca level is < 0.9 mmol/l the treating doctor will be informed so that a rescue dose of 0.5ml/kg of calcium gluconate 10% can be given if appropriate.
  - If the patient subsequently meets the criteria for intubation, or the PaCO2 rises >45 mmHg, the intubation steps described above will be acted upon.
- Any further interventions will be performed according to the Vietnamese MOH guidelines for HFMD management

**APPENDIX 7:**

PATIENT INFORMATION SHEET & INFORMED CONSENT FORM

MAGNESIUM SULPHATE FOR SEVERE HAND FOOT AND MOUTH DISEASE IN VIETNAM

Your child is being invited to take part in a research study of Hand Food and Mouth Disease (HFMD) treatment because your child is showing early signs of complications. Your child’s participation in this study is entirely your choice. Please read this information sheet carefully or have someone read it to you. Please ask the study staff to explain any information that you are not sure about.

**What is the reason for doing the study?**

HFMD is becoming more common in Vietnam. HFMD is caused by a virus. Most children who get HFMD recover without treatment. Some children get severe complications affecting the brain, at first causing the heart rate and blood pressure to increase, and later causing problems with breathing and shock. At the moment there are no specific drugs or vaccines available to prevent or treat HFMD. This research study will test if a well-known drug called **magnesium sulphate** works to prevent severe disease. Because of the way that this drug is used to treat similar diseases, we think that this medicine may control the problems with the heart and blood pressure, and reduce the risk of the child going on to develop shock or breathing problems. This treatment works well to prevent these severe complications in other diseases. The treatment does not kill the virus, but may reduce the chances of severe effects of the virus. The way to find out if magnesium sulphate will help patients with HFMD is by comparing patients who get this treatment to patients who do not. This is what we will do in this study.

This project is organized by the {Hospital Name} and Oxford University and it has been approved by the Oxford Tropical Research Ethics Committee, the {Hospital Name} Ethics Committee and the Viet Nam Ministry of Health. About 190 patients in hospitals in Viet Nam will be a part of this study.

**What will happen if your child participates in the study?**

Whether or not your child takes part in the study, your child will be given standard treatment for HFMD according to the guidelines of the Viet Nam Ministry of Health.

If you agree that your child will be in the study, s/he will be given one extra medicine, which is the study treatment, in addition to the regular treatment. The study treatment is given through a drip in the arm that is used for the regular treatment in these children. The treatment is given for 3 days and the child is monitored very closely. Half of the patients in the study will receive magnesium sulphate as the study treatment; the other half will receive an identical placebo (mixture that looks the same, but has no drug inside). This will allow us to know the effect of magnesium sulphate compared to standard treatment. Which patients receive magnesium sulphate or placebo is decided by chance, and there is an equal chance (50:50) that your child will receive either drug. The purpose of the placebo is so that neither the patients nor the doctors can tell if they are receiving magnesium sulphate. This is called “a double blind placebo controlled trial” and is the best way to study a new treatment, because it gives the most reliable answer regarding which treatment is best.

As a part of regular care, your child will have blood and urine samples and swabs taken to diagnose and monitor their disease. S/he may also have fluid taken from his/her back. This is done in all patients whether or not they enter the study. Doctors will carefully monitor your child, including tests on blood and urine to determine how the drugs are working and to ensure they are not causing any problems. Blood tests would normally be done daily (or more if needed). For this study we would like to collect blood one extra time during the first day. The maximum amount of blood that could be taken for research tests is 5 ml for first day, 2-3 ml for the next three days. It is necessary that your child stays in hospital for at least 3 days, and more if they have not yet recovered.

When your child is ready to discharge, doctors will do a neurological examination on your child. A trained psychologist or teacher will play with your child using toys and language games to assess your child’s physical and language skills. It will take up to 2 hours to complete. The assessor will discuss the results with you and give you a copy of the results if you want to share them with anyone. If your child is not well enough to do this assessment on the day of discharge, you will be asked to return to the hospital one week later for the assessment.

If a child has been very sick sometimes they take several weeks or months to recover, and are not able to do all these tests at the early outpatient visit. So we also ask you to bring your child for a follow-up visit 6 months after the study started. At this visit your child will be examined by a doctor and will have a second neurological assessment.

**What happens to the samples taken?**

Samples taken will be used to give results that help your doctor decide how to care for you. Some extra tests will be done for the research study to find out how the study treatment is working. Some of the samples will be stored to do tests on HFMD and response to infection in the future. Tests that cannot be done in Viet Nam will be sent out of the country for testing. You can decide if you agree to this by marking the last page of this form.

# What are the possible risks of the study?

Patients will have small additional volumes of blood taken for research purposes. This volume will not affect the health of your child, but if they need one additional needle poke there is a small risk of bruising and infection. The study treatment used is generally considered to be safe, even in newborns, with only minor side effects such as drowsiness or sleepiness – this may be a benefit for some children with HFMD who have jerks. Other side effects have been shown when higher doses of the drug are used, these include slowed breathing, low blood pressure and stomach cramps. Your child will be monitored carefully to ensure they have a controlled amount of treatment which has a low rate of side effects.

In the unlikely event that your child’s health is worse as a result of this study, the study organizers have insurance to cover their care.

**What are the possible benefits of the study?**

This study may not have a direct benefit to your child as it is not yet known if the treatment being tested will improve the health of patients with HFMD. The results from this study will help determine the best way to treat patients with this disease in the future.

If your child participates, the hospital costs for diagnosis and treatment of HFMD will be covered by the study from the time that your child enrolls until discharge, not including treatment prescribed before the study starts. All tests done for the study will also be paid by the study organizers. When you return to the hospital for scheduled follow-up visits, we will reimburse you a travel fee of about 150,000 VND if you live in Ho Chi Minh City, and more if you live further. The study staff can tell you exactly how much.

**Research participation**

Being in a research study is your decision. Your child will receive the best available care whether or not they agree to participate. If you enroll your child to the study, you may withdraw him/her for any reason, at any time. If this occurs, the child will be managed in accordance with standard clinical care guidelines.

**Confidentiality:**

All information about your child will be kept confidential. Your child’s medical records will be reviewed in strict confidence by those who are working on this study and may also be reviewed by the ethics committees and health authorities reviewing the study. Your child’s name will not be used on any of the study documents or on the stored samples or in any reports or publications about this study.

# Questions:

If you have any other questions about the study please contact {Dr. Name} at {Dr. Number}. If you have questions about being a participant in a research study please contact the Research Ethics Board of {Hospital Name} at {phone number} or the Research Office at the Hospital for Tropical Diseases at 3924 1983.

**INFORMED CONSENT FORM**

MAGNESIUM SULPHATE FOR SEVERE HAND FOOT AND MOUTH DISEASE IN VIETNAM

- I have read the information given to me and freely agree for my child to be in this study. I have also had a chance to discuss it with the study staff. I will be given a copy of this form to keep.
- I have been told about the risks and benefits. I got answers that I could understand to all my questions.
- I consent to study staff collecting information about my child’s health information, including information already collected from when they were admitted to hospital, and using this information for future medical research about HFMD and infection
- I understand that I can withdraw my child from the study at any time. Stopping will not affect my child’s future care. If I decide to stop the study, I agree that the information collected up that point can be used.
- I agree that samples may be stored for future testing and that this testing can take place outside of Viet Nam

PARTICIPANT 02EI [__][__]-[__][__][__]

By signing/marking my name here, I confirm what is written above.

| Patient’s name: | x ___________________________________________ | | |
| --- | --- | --- | --- |
| **Signature of Person Giving Consent:**  x_______________ | Print Name:  x_____________ | Relationship to Participant:  x______________ | Date of Signature:  ____/____/_____ |

I, the undersigned, have fully explained the relevant information of this study to the signatory above and will provide her/him with a copy of this signed and dated informed consent form.

| x___________________ | x_______________ | ___/____/_____ |
| --- | --- | --- |
| **Study Staff Signature** | Print Name | Date of Signature |

**If the person giving consent cannot read the form themselves, a witness must be present and sign here:**

This form was read accurately to the volunteer, all questions from the volunteer were answered and the volunteer has agreed to take part in the research.

| x_______________ | x_____________________ | _____/______/_____ |
| --- | --- | --- |
| **Witness Signature** | Print Name | Date of Signature |

**Genetic studies**

These may include tests on your genetic code to try and understand why some people get sick from this disease, while others do not. This will involve studying the DNA from your cells**.** If you agree, your child’s DNA will be tested. Tests that cannot be done in Viet Nam will be sent out of the country for testing. Some people may consider tests on their genetic code to be an invasion of privacy. Results will be made available to other researchers but your child will not be identified and no one will know whose genetic information it is. These processes are optional, and your child’s inclusion in the study is independent of this. If you do not agree, your child is still in the study.

By signing/marking my name here, I confirm what is written above.

| Patient’s name: | x ___________________________________________ | | |
| --- | --- | --- | --- |
| **Signature of Person Giving Consent:**  x_______________ | Print Name:  x_____________ | Relationship to Participant:  x______________ | Date of Signature:  ____/____/_____ |

**12-36 hours after consent was given:**

I, the undersigned, have reviewed the study procedures, risks, right to withdraw and alternatives to participation with the parent/guardian of the participant.

| x___________________ | x_______________ | ___/____/_____ |
| --- | --- | --- |
| **Study Staff Signature** | Print Name | Date of Signature |

**APPENDIX 8: STUDY SCHEDULE FOR ALL PLANNED INVESTIGATIONS**

|  | Screening | Enrolment^a^ / D 1 | | | | D 2 | D 3 | D 4 | D 5+ | Discharge | Discharge +7 days | M6 |
| --- | --- | --- | --- | --- | --- | --- | --- | --- | --- | --- | --- | --- |
| Hour from admission | T-x | T-1 | T (-1/2) | T0 | T12 | T24 | T48 | T72 | T96+ |  |  |  |
| **Clinical activities** | | | | | | | | | | | | |
| Screening assessment |  |  |  |  |  |  |  |  |  |  |  |  |
| Informed consent |  |  |  |  |  |  |  |  |  |  |  |  |
| History and physical assessment |  |  |  |  |  |  |  |  |  |  |  |  |
| Randomization assign and study drug preparation |  |  |  |  |  |  |  |  |  |  |  |  |
| Study drug administration |  |  |  |  |  |  |  |  |  |  |  |  |
| Patient assessment |  |  |  |  |  |  |  |  | b | c | (c) | c |
| Recording/ downloading hemodynamic data |  | d | d | d/e | d/e | d/e | d/e | d/e |  |  |  |  |
| **Research blood test** | | | | | | | | | | | | |
| Chemistries |  | 0.5 ml |  | 1.5 ml | 1 ml | 2 ml | 2 ml | 2 ml |  |  |  |  |
| **Specialized additional research blood test** | | | | | | | | | | | | |
| Serum catecholamine |  |  |  | 2 ml |  | 2 ml | 2ml | 2 ml |  |  |  |  |
| Cytokine profile |  |  |  | 1 ml | 1 ml | 1 ml |  |  |  | 1 ml |  |  |
| Serology | 1 ml |  |  |  |  |  |  |  |  | 1 ml | |  |
| **Research procedures** | | | | | | | | | | | | |
| ECG recording^f^ |  |  |  |  |  |  |  |  |  |  |  |  |
| Arterial catheterization |  |  |  |  |  |  |  |  |  |  |  |  |
| Blood draw |  |  |  |  |  |  |  |  |  |  |  |  |
| Diagnosis swabs |  |  |  |  |  |  |  |  |  |  |  |  |
| **Additional procedure** | | | | | | | | | | | | |
| Brain MRI |  |  |  |  |  |  |  |  | Only when stable | |  |  |
| **Urine-Research Laboratory** | | | | | | | | | | | | |
| Urine Catecholamine |  |  |  |  |  |  |  |  |  |  |  |  |
| Total amount of blood | 1 ml | 7 ml  13 ml | | | | 5 ml | 4 ml | 4 ml |  | 2 ml | |  |

Note that additional clinical investigations will be performed as necessary when indicated clinically

Note: a – Enrollment stage should be done as fast as possible: 30 minutes to take informed consent and check exclusion criteria, 30 minutes for drug administration.

b- Clinical assessment will be carried out daily until discharge

c- Neurological outcome and development will be assessed in this stage. Patients unable to be assessed at discharge will be invited to attend for assessment after 7 days.

d- Standard hemodynamic data will be recorded/downloaded and stored in PC

e- Advanced hemodynamic data will be downloaded from LiDCOrapid and stored in PC

f: Standard ECG recording will be performed daily and whenever an abnormality is noted on the monitor

## APPENDIX 9: LABORATORY SCHEDULE (CLINICAL AND RESEARCH INVESTIGATIONS)

|  | D1 | | D2 | D 3 | D4 | Discharge or D+7 days |
| --- | --- | --- | --- | --- | --- | --- |
| (Hour) | T0 | T 12 | T 24 | T 48 | T 72 |  |
| Na, K, Mg, Ca, Cl | ✓ |  | ✓ | ✓ | ✓ |  |
| Mg, Ca |  | ✓ |  |  |  |  |
| Blood Gases | ✓ |  | ✓ | ✓ | ✓ |  |
| Creatinine | ✓ |  | ✓ | ✓ | ✓ |  |
| CK MB | ✓ |  | ✓ | ✓ | ✓ |  |
| Troponin I | ✓ |  | ✓ | ✓ | ✓ |  |
| Plasma catecholamines | ✓ |  | ✓ | ✓ | ✓ |  |
| Urine catecholamines | ✓ |  | ✓ | ✓ | ✓ |  |
| Cytokines | ✓ | ✓ | ✓ |  |  | ✓ |
| Serology | ✓ |  |  |  |  | ✓ |
| **Amount of blood**  **(ml)** | 5 | 2 | 5 | 4 | 4 | 2 |
| **Total blood volume** | 7 ml | | 5 ml | 4 ml | 4 ml | 2 ml |

The amount of blood taken for the various tests is as follows:

Electrolytes (including Mg, Ca), Creatinine, CKMB, Troponin I, and Glucose: 1,5 mls

Plasma Mg/Ca alone: 1 ml

Arterial blood gas: 0.5 ml

Plasma catecholamines: 2 mls

Cytokines, serology: 1-2 mls

Blood sugar will also be checked on the ward 6 hourly on a drop of blood from the arterial line

**APPENDIX 10:** **Modified Adverse Events Grading in HFMD trial [**[**13**](#_ENREF_13)**]**

|  | Grade | | | | |
| --- | --- | --- | --- | --- | --- |
| Adverse Event | 1 | 2 | 3 | 4 | 5 |
| 1. General | | | | | |
| Fever after enrolment (core T) | 39 - < 40^9 C^ | 40-41^0^C without any new CNS problem | 40-41^0^C with new CNS problem | > 41 ^0^C with new CNS problem or >42^0^C for over 1 hour |  |
| Flushing | Asymptomatic, no intervention needed | Moderate, requiring symptomatic Rx only, eg antihistamines | Associated with hypotension and/or tachycardia | - | - |
| Mouth or Skin lesion | New lesions noted | - | - | - | - |
| Profuse sweating | Local | General | - | - | - |
| Mottled skin | Local | General | - | - | - |
| 2. Gastrointestinal disorders | | | | | |
| Vomiting | 1 - 2 times per day | 3 - 5 times per day | >= 6 times per day | - | - |
| Diarrhea | 1 - 3 loose stools per day | 4 – 6 loose stools per day | >=7 loose stools per day | Life-threatening with signs of dehydration. | Death |
| Liver palpable | Asymptomatic | Painful | - | - | - |
| Upper GI Bleeding | Occasional streaks of blood in vomit | Persistent blood but no clinical problems | Blood in vomit, needing Tx but not urgent | Life-threatening and urgent transfusion needed | Death |
| Lower GI Bleeding | Occasional streaks of blood in stool | Persistent blood but no clinical problems | Blood in stool, needing Tx but not urgent | Life-threatening and urgent transfusion needed | Death |
| 3. Cardiac disorders | | | | | |
| Cardiac arrest | - | - | - | Life-threatening, and urgent intervention indicated | Death |
| Atrioventricular block complete | - | No hemodynamic effect and resolves after stopping study drug | Any hemodynamic effects, even if these resolve after stopping study drug. Advice from cardiologist, but not urgent | Life-threatening with hemodynamic compromise, and requiring immediate intervention eg urgent pacing wire | Death |
| Atrioventricular block first degree | - | Asymptomatic, no intervention | - | - | - |
| 2^nd^ Degree (Mobitz Type I or II) atrioventricular block |  | Asymptomatic, no intervention | Hemodynamic effects, requiring advice from cardiologist, not urgent | Life-threatening with hemodynamic compromise, and requiring immediate intervention | Death |
| QT interval (corrected for heart rate)* | QTc 450 - 480 ms | QTc 481 - 500 ms | QTc >= 501 ms on at least two separate ECGs | QTc >= 501 or >60 ms change from baseline and Torsade de pointes or polymorphic VT or other signs/symptoms of serious arrhythmia | Death |
| Other serious rhythm disturbance (on the monitor or a formal ECG) | - | Occasional atrial or ventricular ectopics, no intervention | Persistent atrial or ventricular ectopics, causing hemodynamic effects but resolving after withdrawal of study drug. AF not causing hemodynamic compromise or thrombotic / embolic complications. | Atrial or ventricular ectopics not responding to withdrawal of study drug. VT or VF. AF if associated with any thrombotic / embolic complication. | Death |
| Hypotension  (Systolic BP <70+2Xage) |  | Transient hypotension: close observation, no intervention, resolves within 15 mins | Medical intervention required but not urgent | Life-threatening, and urgent intervention indicated | Death |
| Hypertensive emergency | - | - | - | Life-threatening, and urgent intervention indicated | Death |

*: Corrected QT (QT_C_) = Bazett's Formula = QT Interval / √ (RR interval)  RR Interval = 60/HR

|  | Grade | | | | |
| --- | --- | --- | --- | --- | --- |
| Adverse Event | 1 | 2 | 3 | 4 | 5 |
| 4. Respiratory disorders | | | | | |
| Hypoxia | - | Intermittent O2 saturation <92%, brief, no respiratory symptoms | Persistent O2 saturation <92%, despite nasal O2, but resolves in < 60 minutes | Decreased O2 saturation <92 %, failing to improve with nasal O2, and persisting for > 60 minutes requiring ventilator support . | Death |
| Irregular breathing | Irregular breathing occurring transiently and no O2 indicated | Irregular breathing is persistent but SpO2 >92% when checked in air. | Irregular breathing and SpO2 < 92% without O2 | Life-threatening, needing urgent intervention, eg intubation or ventilator support | Death |
| Stridor | - | Stridor occurs transiently but SpO2 >92% when checked in air. | Stridor occurs and SpO2 <92% without O2 | Life-threatening, needing urgent intervention, eg intubation or ventilator support | Death |
| Respiratory retractions | - | Retractions occur transiently but SpO2 >92% when checked in air. | Retractions occur and SpO2 <92% without O2 | Life-threatening, needing urgent intervention, eg intubation or ventilator support | Death |
| Apnea/Cheyne Stokes/ Gasps | - | - | - | Life-threatening, needing urgent intervention, eg intubation or ventilator support | Death |
| Pulmonary edema |  |  | Severe dyspnea or dyspnea at rest; O2 indicated; | Life-threatening respiratory compromise; urgent intervention or intubation with ventilator support indicated | Death |
| 5. Nervous system disorders | | | | | |
| Coma | GCS ≥ 14 | 11 ≤ GCS ≤ 13 | 9 ≤ GCS ≤ 10 | GCS ≤ 8 | Death |
| Convulsions |  | Brief (< 1 minute) generalized seizure | Multiple seizures requiring drug Rx but not respiratory support | Prolonged repetitive seizures, poor response to treatment, requiring respiratory support (CPAP or IPPV) | Death |
| Pupils reactive | - | - | Unreactive or > 3mm in one eye | Unreactive or > 3mm in both eyes |  |
| Headache | Mild pain, no medication needed | Moderate pain requiring paracetamol intermittently | Severe pain, requiring regular or continuous paracetamol. | - | - |
| Lethargy | Intermittent reduced alertness and awareness (not related to a convulsion) | Persistently reduced alertness and awareness for several hours | - | - | - |
| Irritability | Intermittent | Persistent | - | - | - |
| Myoclonic jerks | ≤ 2 times/ day | 3 – 10 times/ day | > 10 times /day | - | - |
| Ataxia/tremor | Intermittent | Persistent | - | - | - |
| Nystagmus / eye wandering | Intermittent | Persistent | - | - | - |
| Diminished deep tendon reflexes (DTRs) | Asymptomatic, reduced but still present | Complete loss of DTRs, no resp. distress | Complete loss of DTRs, with resp. distress | - | - |
| Limb paralysis (severity assessment at discharge) | Minor weakness, only detectable on examination | Partial weakness of 1 limb, but able to function (age appropriate) with support | Partial weakness of >1 limb, but able to function (age appropriate) with support | Profound weakness, dependent on support from carers |  |
| Cranial nerve paralysis (severity assessment at discharge) | - | Observation only, no clinical effects | With clinical effects, eg. Swallowing difficulty, medical management only | With severe clinical consequences requiring invasive management eg. Tracheostomy | - |

|  | Grade | | | | | | | |
| --- | --- | --- | --- | --- | --- | --- | --- | --- |
| Adverse Event | 1 | 2 | | 3 | | 4 | 5 | |
| 6. Renal disorders | | | | | | | | |
| Reduced urine output | - | Transient reduction in urine output, improving within 4 hours without Rx |  | |  | | | Death |
| Haematuria | - | Visible blood but no clinical problems | Needing Tx but not urgent | | Life-threatening and urgent transfusion needed | | | Death |
| Urinary retention | Temporary, responding to physical stimulation of bladder | Persistent requiring catheterisation |  | |  | | |  |
| 7. Other | | | | | | | | |
| Cough, coryza, conjuncitivitis | Mild symptoms, no treatment needed | - | - | | - | | | - |
| Abdominal Pain | Mild pain, no medication needed | Moderate pain requiring paracetamol intermittently | Severe pain, requiring regular or continuous paracetamol. | |  | | | - |
| Abdominal distension | Mild, visible but asymptomatic | Causing some minor distress but no intervention needed | Causing distress sufficient to need rectal catheter insertion | | Resulting in perforation | | | - |
|  |  |  |  | |  | | |  |

| Grade | | | | | |
| --- | --- | --- | --- | --- | --- |
| Laboratory Abnormalities | 1 | 2 | 3 | 4 | 5 |
| Acidosis | pH <7.37, but >=7.3 | - | pH <7.3 | Life-threatening consequences | Death |
| Alkalosis | pH >7.45, but <=7.5 | - | pH >7.5 | Life-threatening consequences | Death |
| Hb | Hemoglobin (Hgb):  6monts-2yrs: 10 - <10.5 g/dl  >2 yrs: 10.0 - < 11.5 g/dL; | Hgb <10.0 - 8.0 g/dL; | Hgb <8.0 g/dL; | Life-threatening consequences; urgent intervention indicated | Death |
| Platelet count decreased | <LLN - 75,000/mm3; | <75,000 - 50,000/mm3; | 50,000 - 25,000/mm3; | < 25,000/mm3; |  |
| Cardiac Troponin I increased* | (>ULN - 2.5 x ULN) | >2.5 x ULN - 5 x ULN | >5 x ULN - 10 x ULN | 10 x ULN | - |
| Before 11Mar2015: ULN: 0.3 ng/l | > 0.3- 0.75 | > 0.75- 1.5 | >1.5- | > 3 |  |
| From 11Mar2015: ULN: 16 pg/l | >16- 40 | > 40- 80 | >80 – 160 | > 160 |  |
| CK-MB  ULN: 24 UI/l | >ULN - 2.5 x ULN  (> 24- 60) | >2.5 x ULN - 5 x ULN  (> 60-120) | >5 x ULN - 10 x ULN  (>120 – 240) | 10 x ULN  (>240) |  |
| Creatinine increased  ULN: <4yr : 42.2, / 4-10yr : 52.2 >10-14 yr: 77.8 umol/l | >ULN -1.5 x ULN | >1.5 - 3.0 x ULN | >3.0 - 6.0 x ULN | >6.0 x ULN | - |
| Hyponatremia  LLN: 135 mmol/l | <135 - 130 mmol/L | - | <130 - 120 mmol/L | <120 mmol/L | Death |
| Hypernatremia  ULN: 145 mmol/l | >145 - 150 mmol/L | >150 - 155 mmol/L | >155 - 160 mmol/L; | >160 mmol/L | Death |
| Hyperkalemia | >5 - 5.5 mmol/L | >5.5 - 6.0 mmol/L | >6.0 - 7.0 mmol/L; | >7.0 mmol/L | Death |
| Hypokalemia | <3.5 - 3.0 mmol/L | <LLN - 3.0 mmol/L; | <3.0 - 2.5 mmol/L; | <2.5 mmol/L; | Death |
| Hypermagnesemia | >1 -1.23 mmol/L | ->1.23 – 2.5 mmol/L | >2.5 – 3.3 mmol/L. | >3.30 mmol/L; | Death |
| Hypocalcemia  2.2-2.7 mmol/l  (~ ionized calcium: 1.1-.135) | Serum calcium of <2.2 - 2 mmol/L | Serum calcium of <2.0 - 1.8 mmol/L | Serum calcium of <1.8 - 1.5 mmol/L | Serum calcium of <1.5 mmol/L | Death |
| Hypoglycemia | <60 - 55 mg/dL or  < 3.33 – 3.05 mmol/l | <55 - 40 mg/dL or  < 3.05 – 2.2 mmol/l | <40 - 30 mg/dL or  < 2.2 – 1.66 mmol/l | <30 mg/dL or  < 1.66 mmol/l | Death |
| Hyperglycemia | Fasting glucose value >ULN -  160 mg/dL; or > - 8.88 mmol/l | Fasting glucose value >160 - 250 mg/dL  Or > 8.88 – 13.88 mmol/l ; | >250 - 500 mg/dL;  or > 13.88 – 27.8 mmol/l | >500 mg/dL;  or > 27.8 mmol/l | Death |
|  | | | | | |
|  | | | | | |

**REFERENCES FOR APPENDICES**

1. Anonymous. Vietnames MOH Guideline for HFMD management. Hà nội2012.

2. National High Blood Pressure Education Program Working Group on High Blood Pressure in Children and Adolescents: The Fouth Report on the Diagnosis, Evaluation, and Treatment of High Blood Pressure in Children and Adolescents. Pediatrics. 2004;114:555.

3. Flynn JT. Management of hypertension in infants. UpTodate. 2012;19(2).

4. Ewa Otto-Buczkowska TD, Urszula Mazur. Alterations of blood glucose homeostasis in critically ill children – hyperglycemia. Endokrynologia, Diabetologia i Choroby Przemiany Materii. 2007;13(1):4.

5. Smyth RM SP, Armstrong N and Duley L. Magpie Trial in the UK: methods and additional data for women and children at 2 years following pregnancy complicated by pre-eclampsia. BMC Pregnancy and Childbirth. 2009;9.

6. Ho JJ RG. Magnesium sulfate for persistent pulmonary hypertension of the newborn. The Cochrane Library. 2010(3).

7. D K L Cheuk TCHC, S L Lee. A meta-analysis on intravenous magnesium sulphate for treating acute asthma. Arch Dis Child. 2005;90:74–7.

8. Tolsa J.F CJ, Sekarski N. , Payot M ., Micheli J.L , Calame A. Magnesium sulphate as an alternative and safe treatment for severe persistent pulmonary hypertension of the newborn. Archives of Disease in Childhood 1995;72:F184-F7.

9. Thwaites CL, Yen LM, HT L, TT T, Thwaites GE, Stepniewska K et al. Magnesium sulphate for treatment of severe tetanus: a randomised controlled trial. . Lancet. 2006;368(9545).

10. Rowe BH BJ, Bourdon C, Bota G, Blitz S, Camargo CA. Magnesium sulfate for treating exacerbations of acute asthma in the emergency department (Review). The Cochrane Library. 2009(3):25.

11. Agus ZS. Symptoms of hypermagnesemia. In: Goldfarb S, editor. UpTodate. 20.1 ed: Wolters Kluwer Health; 2013.

12. Lexicomp I. Magnesium sulfate: Pediatric drug information. vol Uptodate. 2012.

13. Common Terminology Criteria for Adverse Events (CTCAE). version 4.03 ed: National Cancer Institute (National Institutes of Health); 2010. p. 196.
